# Maternal overnutrition during critical developmental periods leads to different health adversities in the offspring: relevance of obesity, addiction and schizophrenia

**DOI:** 10.1038/s41598-019-53652-x

**Published:** 2019-11-21

**Authors:** Gitalee Sarker, Kathrin Litwan, Rahel Kastli, Daria Peleg-Raibstein

**Affiliations:** 1 Department of Health Sciences and Technology, ETH Zurich, Schwerzenbach, 8603 Switzerland; 2 Present Address: Department of Physiology, Anatomy and Genetics University of Oxford, Sherrington Building, Parks Road, OX1 3PT, Oxford, United Kingdom

**Keywords:** Neuroscience, Diseases of the nervous system

## Abstract

Maternal overnutrition during sensitive periods of early development increases the risk for obesity and neuropsychiatric disorders later in life. However, it still remains unclear during which phases of early development the offspring is more vulnerable. Here, we investigate the effects of maternal high-fat diet (MHFD) at different stages of pre- or postnatal development and characterize the behavioral, neurochemical and metabolic phenotypes. We observe that MHFD exposure at pre-conception has no deleterious effects on the behavioral and metabolic state of the offspring. Late gestational HFD exposure leads to more prominent addictive-like behaviors with reduced striatal dopamine levels compared to early gestational HFD. Conversely, offspring exposed to MHFD during lactation display the metabolic syndrome and schizophrenia-like phenotype. The latter, is manifested by impaired sensory motor gating, and latent inhibition as well as enhanced sensitivity to amphetamine. These effects are accompanied by higher striatal dopamine levels. Together, our data suggest that MHFD exposure during specific stages of development leads to distinct neuropathological alterations that determine the severity and nature of poor health outcome in adulthood, which may provide insight in identifying effective strategies for early intervention.

## Introduction

The escalating growth of obesity is a global threat to the health and prosperity irrespective of age and sex. At present, approximately 36.5% men and 38% women worldwide are classified as obese^1^. It is even more alarming that the prevalence of maternal obesity has risen dramatically over the past two decades^2^. A recent survey has reported that around 27% women of reproductive age are overweight and 37% are obese in the United States with a similar pattern in Europe^3^.

Maternal obesity or overnutrition is one of the major determinants of poor pregnancy outcome^4^. Obesity during pregnancy negatively affects the embryonic and fetal growth^5^ but it may also have long lasting influences on the health of the offspring. In this context, the interaction between the in utero milieu, fetal, perinatal development and the increased risk of adult onset diseases has been highlighted in recent years^6^. This theory, also known as the ‘Developmental Origin of Health and Disease’, postulates that the fetus makes permanent adaptations in the homeostatic system in response to suboptimal intrauterine conditions leading to increased risk to develop non-communicable diseases later in life^7^.

Emerging data from epidemiological studies have revealed the association between maternal obesity and the increased risk of obesity^8^, cardio metabolic diseases^9^ and mental disorders^10–12^ in the progeny during childhood, adolescence and adulthood. However, human studies are limited in their ability to determine the causal link between maternal obesity and the increased disease risk in the offspring due to several confounding factors including shared genetics, postnatal feeding habits and parental lifestyles. In addition, women who gain weight prior to conception remain obese throughout pregnancy and breastfeeding, which makes it difficult to determine the independent contribution of obesity during these different phases on the health of the progeny. Therefore, studies in experimental animals are invaluable in identifying the specific link between maternal obesity and adverse health outcomes in the offspring as well as exploring the critical windows of vulnerability during development.

Indeed, studies in rodents demonstrate that maternal HFD (MHFD) exposure before and throughout pregnancy and lactation induces adiposity, hyperglycemia, insulin resistance and dyslipidemia associated with pancreatic beta cell dysfunction as well as altered hepatic gene expression related to glucose and lipid metabolism in the offspring^13–15^. Recently, MHFD was shown to affect brain development and behaviors of the offspring such as impaired cognitive functions^16–20^, increased emotionality^21^, addiction-like behaviors and motivational aspects^22–25^.

We have shown in our previous studies that mice exposed to a MHFD for a total period of 9 weeks (3 weeks prior to conception, 3 weeks during gestation and 3 weeks during lactation) show an obesogenic and metabolic syndrome like traits and a wide range of behavioral alterations such as increased anxiety^21^, enhanced sensitivity to palatable foods and drugs of abuse^22^, and age dependent cognitive deficits^18^. It is now crucial to identify the specific developmental time window, which is most vulnerable to such programming effects of MHFD exposure. This will advance potential intervention at a specific period of development for the prevention and management of disease process. Thus far, only a handful of studies in rodents have investigated the effects of MHFD exposure during specific periods of gestation and lactation. For example, differential effects on offsprings’ metabolic programming following a pre-pregnancy short-term HFD exposure^26^ or specific periods of gestation^27^ have been explored. MHFD exposure during lactation induces adiposity, insulin resistance and disruption of hypothalamic development in the offspring, although the mechanisms for such effects need to be further evaluated^28,29^. To date, few studies attempted to investigate how MHFD exposure at specific stages of development may lead to progression of a wide variety of mental disorders in the offspring. The present study therefore, aims to investigate the independent effects of MHFD exposure during four different developmental time windows: (i) preconception (PC; 3 weeks prior to mating), (ii) early gestation (EG; G0-G11) and (iii) late gestation (LG; G12-G21) as well as (iv) lactation (Lact; postnatal day 1–21), on behavioral and metabolic programming of the offspring. Furthermore, we have assessed dopamine (DA) and its metabolites levels in brain regions related to the central reward system from adult offspring exposed to MHFD during different periods of development and their controls. We have focused on the mesolimbic dopaminergic system because of its central role in the pathogenesis of obesity^30^ as well as neuropsychiatric disorders^31^ and its special developmental trajectory during prenatal and postnatal periods^32^.

## Results

### The effects of preconception MHFD exposure

**Figure 1:**
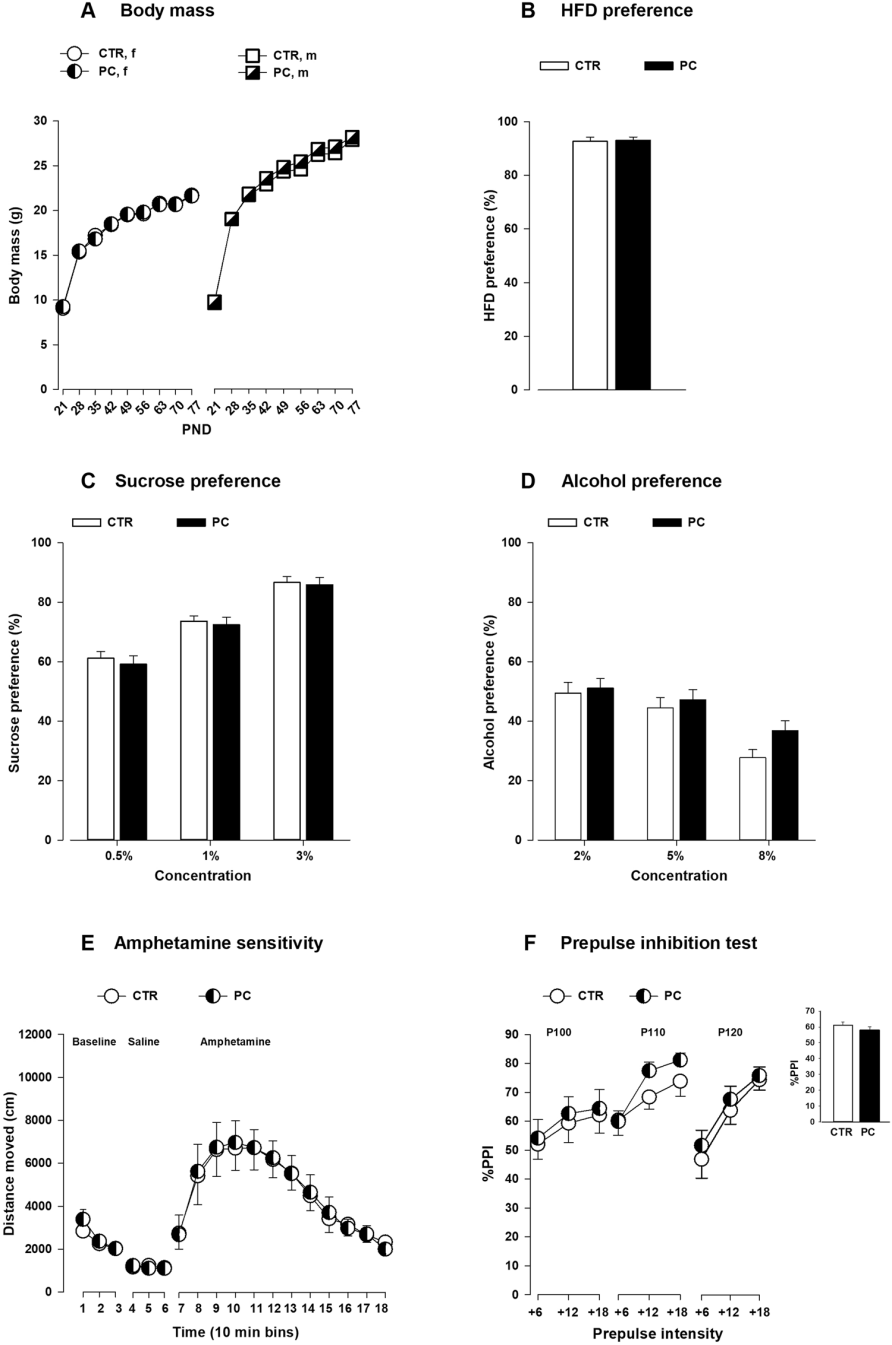
Effects of maternal HFD exposure prior to conception on physiology and behavior of the offspring. (**A**) The line graph shows the body mass measured weekly from PND 21–77. N = (46 m, 46f) CTR and N = (24 m, 26f) PC. **(B)** HFD preference test: The bar graph shows the mean HFD preference in percentage in the PC and CTR offspring. N = (10 m, 10f) per group. **(C)** Sucrose preference test: the bar plot depicts the mean sucrose preference in three different concentrations (0.5%, 1% and 3%). N = (10 m, 10f) per group. **(D)** Alcohol preference test: the bar graph shows the mean alcohol preference in three different concentrations (2%, 5% and 8%). N = (10 m, 10f) per group. **(E)** Amphetamine sensitivity: the line plots show the distance travelled in successive 10 min bins following baseline, saline and amphetamine injection in the open field. N = (5 m, 5f) per group. **(F)** Prepulse inhibition of the acoustic startle reflex: the line plot shows the percent PPI as a function of different pulses (100, 110 and 120 dB) and the corresponding prepulse intensities (+6, +12 and +18 DB above the background 65 dB). The bar plot depicts the comparison of the mean %PPI between the groups. N = (10 m, 10f) per group. All values are mean ± s.e.m. f, female; m, male; PC = preconception; CTR = control; HFD = high-fat diet; PND = postnatal day.

No difference in body mass was observed between the offspring born to preconception HFD and chow fed dams **(**Fig. 1A**)**. Body mass of all offspring gradually increased over 9 weeks as supported by a main effect of PND (F_8,1040_ = 2498.39; *P* < 0.0001). Next, we analyzed the hedonic responses to natural rewards (HFD and sucrose) in the PC and CTR groups. In the HFD preference test, ANOVA revealed a significant main effect of diet (*F*_1, 28_ = 261.39; *P* < 0.0001). The offspring from both groups did not differ in their preference to HFD **(***F*_1, 27_ = 0.02; *P* = 0.89; Fig. 1B**)**. Similarly, in the sucrose preference test, both offspring groups preferred the sucrose solution over water (*F*_1, 28_ = 254.98; *P* < 0.001). No difference in sucrose preference was detected between the groups at all concentrations (*F*_1,28_ = 0.24; *P* = 0.63; Fig. 1C**)**. Further, when offspring were given a free choice to drink from water or alcohol solution, offspring from both groups consumed more alcohol than water as indicated by a main effect of solution (*F*_1,28_ = 33.540; *P* < 0.001). However, no difference was observed in alcohol preference between the groups (*F*_1,28_ = 2.04; *P* = 0.17; Fig. 1D**)**. In the amphetamine sensitivity test, no difference in baseline locomotor activity or following a saline injection was observed between the offspring groups (Fig. 1E**)**. Following a systemic amphetamine challenge, locomotor activity increased in both groups and gradually decreased over the period of 2 hours as was supported by a main effect of 10-min bins (*F*_11,176_ = 49.19; *P* < 0.001). No difference in response to amphetamine was detected between CTR and PC offspring. We further evaluated the effects of preconception MHFD on sensorimotor gating as assessed by prepulse inhibition (PPI) of startle in adulthood. The levels of PPI were increased with increased levels of prepulse intensities in both groups which was supported by a main effect of prepulse intensity (*F*_2,56_ = 29.23; *P* < 0.0001) and a significant pulse intensity × prepulse intensity interaction (*F*_4,112_ = 5.55; *P* < 0.0005). However, no difference was detected in % PPI between PC and CTR offspring for each of the three pulse intensities (Fig. 1F).

### The effects of MHFD during early and late gestation

**Figure 2:**
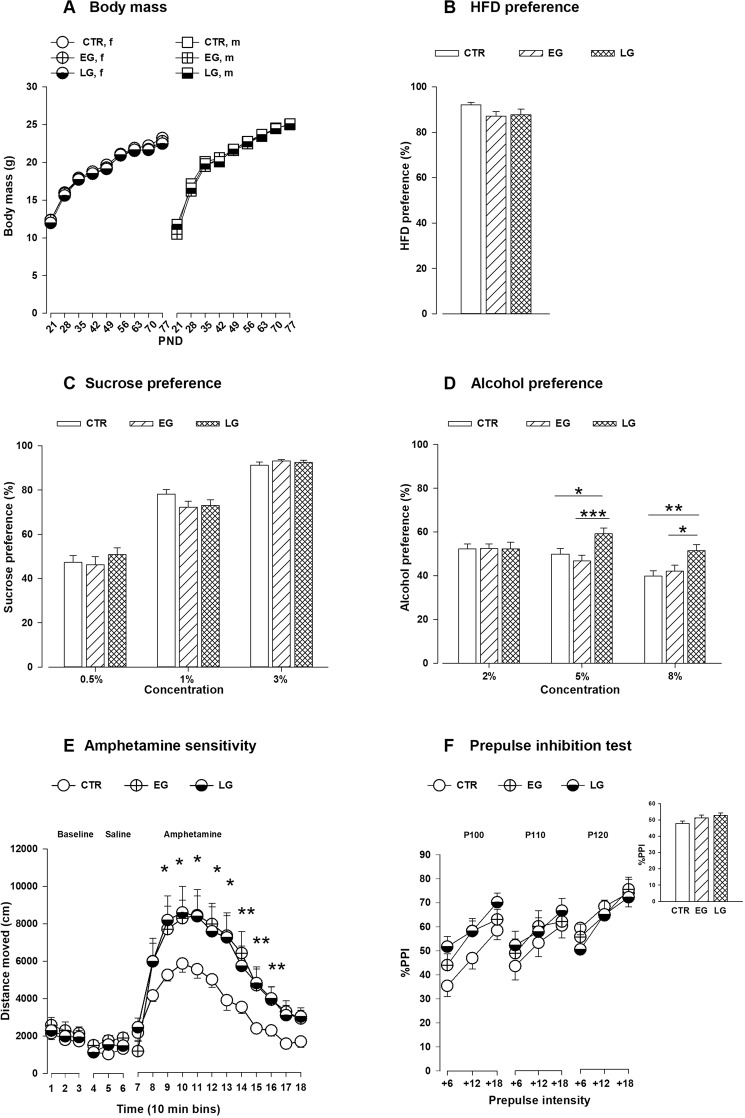
Effects of maternal HFD exposure during gestation on the physiology and behavior of the offspring. (**A**) Body mass of male and female offspring measured weekly from PND 21–77. N = (91 m, 76f) EG; N = (43 m, 37f) LG and N = (32 m, 36f) CTR. **(B)** HFD preference test: the bar graph shows the offspring from all groups prefer HFD to chow. N = (10 m, 10f) per group. **(C)** Sucrose preference test: the bar plot displays the mean sucrose preference in ascending concentrations (0.5%, 1% and 3%) in the different offspring groups. N = (10 m, 10f) per group. **(D)** Alcohol preference test: the bar graph shows the mean alcohol preference in three different concentrations (2%, 5% and 8%). N = (10 m, 10f) per group. **(E)** Amphetamine sensitivity: the line plot shows the locomotor activity in the open field expressed as distance traveled (cm) per 10-min bin during the baseline, following saline administration and following a systemic amphetamine injection. N = (4 m, 4f) per group. **(F)** %Prepulse inhibition of acoustic startle reflex: the line plot shows the percent PPI as a function of different pulses (100, 110 and 120 dB) and the corresponding prepulse intensities (+6, +12 and +18 DB above the background 65 dB). The bar plot depicts the comparison of mean %PPI between the groups. N = (10 m, 10f) per group. All values are mean ± s.e.m. *P < 0.05; **P < 0.001; ***P < 0.0001. f, female; m, male; EG = Early gestation; LG = Late gestation; CTR = control; HFD = high fat diet; PND = postnatal day.

Male and female offspring from all groups gained weight from weaning until adulthood (PND 77) as depicted in Fig. 2A and supported by a main effect of PND (*F*_9,2709_ = 4146.96; *P* < 0.0001). However, no difference in body mass was detected among the offspring from EG, LG and CTR groups **(**Fig. 2A**)**. In the HFD preference test, a greater intake of HFD was observed in all offspring groups and no difference was detected in HFD preference between the offspring groups (*F*_2,54_ = 2.74; *P* = 0.07; Fig. 2B**)**. In the sucrose preference test, all offspring groups displayed increased sucrose consumption as the sucrose concentration increased supported by the main effect of solution (*F*_1,51_ = 287.96; *P* < 0.001) and concentration (*F*_2,104_ = 73.67; *P* < 0.0001). However, no difference was detected between the offspring groups in sucrose preference **(**Fig. 2C**)**. In the alcohol preference test, offspring exposed to HFD during LG preferred more alcohol at higher concentrations compared to both EG and CTR offspring (*F*_2,51_ = 3.65; *P* < 0.03). *Post hoc* analysis revealed that LG offspring consumed more alcohol at higher concentrations compared to both CTR (CTR vs LG: 5% alcohol, *P* < 0.02; 8% alcohol, *P* < 0.003) and EG offspring (EG vs LG: 5% alcohol, *P* < 0.0001; 8% alcohol, *P* < 0.02) (Fig. 2D). In the amphetamine sensitivity test, no difference was observed in the baseline locomotion or in response to a saline injection between the groups. A significant increase in locomotor activity was detected in the gestation HFD groups compared to the CTR (*F*_2,17_ = 3.13; *P* < 0.05) **(**Fig. 2E**)**. *Post hoc* comparisons confirmed that both EG (*P* < 0.02) and LG (*P* < 0.001) offspring showed enhanced locomotor response to amphetamine compared to CTR offspring. In the PPI test, the %PPI increased as a function of higher levels of prepulse intensities in both groups (main effect of prepulse intensity, *F*_2,100_ = 49.64; *P* < 0.0001) as shown in Fig. 2F. However, no difference was observed in %PPI among the different groups for each of the three pulse-prepulse intensities.

### The effects of MHFD during lactation in the metabolic phenotypes of the offspring

**Figure 3:**
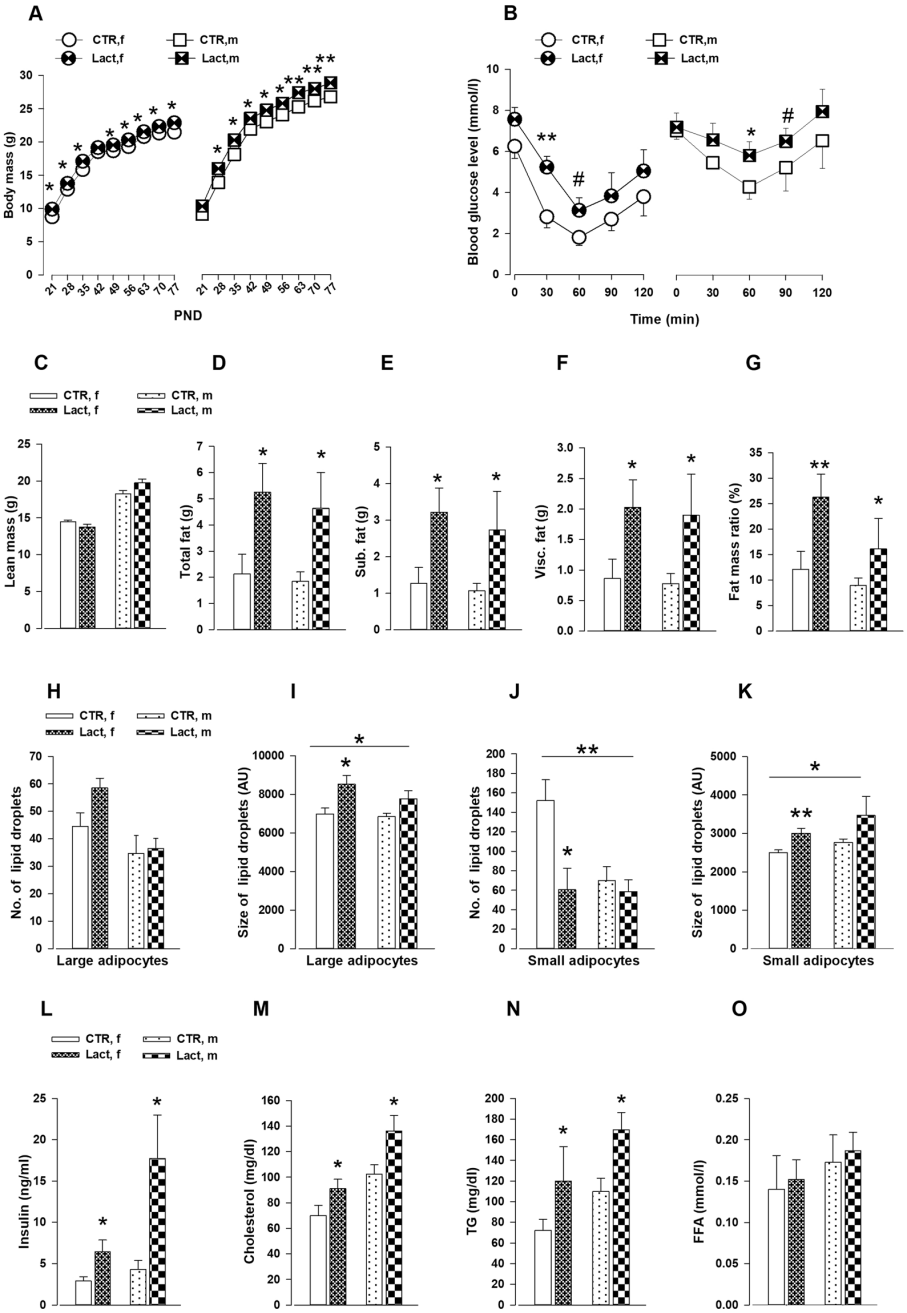
Characterization of the metabolic phenotype of the offspring exposed to maternal HFD during lactation. (**A**) The line plots depict the body mass of both male and female offspring measured from PND 21–77. N = (24 m, 26f) Lact; and N = (19 m, 12f) CTR. **(B)** Insulin tolerance test: the line plots depict the blood glucose levels measured before (0 min) and after a systemic insulin injection at specific time intervals (30, 60, 90 and 120 min). N = (6 m, 6f) per group. **(C–G)** Fat mass distribution: the bar graphs show the lean mass, total fat mass, subcutaneous and visceral adipose tissue content as well as fat mass ratio measured by CT scan. N = (6 m, 6f) per group. **(H–K)** Lipid droplets measurement in visceral adipocytes: the bar graphs show the number and size of the lipid droplets in the large and small adipocytes. N = (6 m, 6f) per group. **(L–O)** Plasma parameters: metabolic profile from blood samples taken from fasted lactation and CTR offspring. N = (6 m, 6f) per group. All values are mean ± s.e.m. *P < 0.05; **P < 0.001; ***P < 0.0001. f, female; m, male; Lact = lactation; CTR = control; PND = postnatal day; Sub. = subcutaneous; Visc. = visceral; TG = triglyceride; FFA = free fatty acid.

Both male and female offspring exposed to MHFD during lactation exhibited increased weight gain from PND 21 to PND 77 compared to CTR offspring (*F*_1,81_ = 12.38; *P* < 0.0008) **(**Fig. 3A**)**. An insulin tolerance test demonstrated that Lact offspring showed higher blood glucose levels than CTR following an i.p insulin injection (*F*_1,20_ = 4.61; *P* < 0.05) **(**Fig. 3B**)**. The CT-scan revealed that Lact offspring displayed increased body fat content (*F*_1,20_ = 4.46; *P* < 0.04), higher fat content in the subcutaneous (*F*_1,20_ = 4.82; *P* < 0.05) and visceral (*F*_1,20_ = 4.50; *P* < 0.05) adipose tissue and higher fat mass ratio (*F*_1,20_ = 6.53; *P* < 0.02) with no difference in the lean body mass (Fig. 3C–G). To support these observations, the lipid droplet size in the visceral and subcutaneous adipose tissue was analyzed **(**Fig. 3H–K**)**. An ANOVA revealed no difference in the number of large lipid droplets in the visceral fat depot (*F*_1,20_ = 1.73; *P* = 0.203). However, the size of the large lipid droplets was significantly bigger in the lactation offspring compared to the CTR (*F*_1,20_ = 6.40; *P* < 0.03). Lact offspring had less number of lipid droplets (*F*_1,20_ = 8.25; *P* < 0.01) in the small adipocytes but the size of the lipid droplets was larger (*F*_1,20_ = 5.39; *P* < 0.04) compared to CTR offspring. In the subcutaneous adipose tissue, neither the lipid droplets number nor their size differed between the groups. Furthermore, significant alterations in the circulating insulin and lipids levels were detected **(**Fig. 3L–N**)**. Lact offspring showed elevated fasted plasma insulin (*F*_1,19_ = 5.08; *P* < 0.05), cholesterol (*F*_1,19_ = 9.13; *P* < 0.008) and triglycerides (*F*_1,19_ = 7.98; *P* < 0.02) levels. No difference in plasma free fatty acid was observed between the groups (*F*_1,19_ = 0.71; *P* = 0.68) **(**Fig. 3O**)**.

### MHFD exposure during lactation affects behavior of the offspring

**Figure 4:**
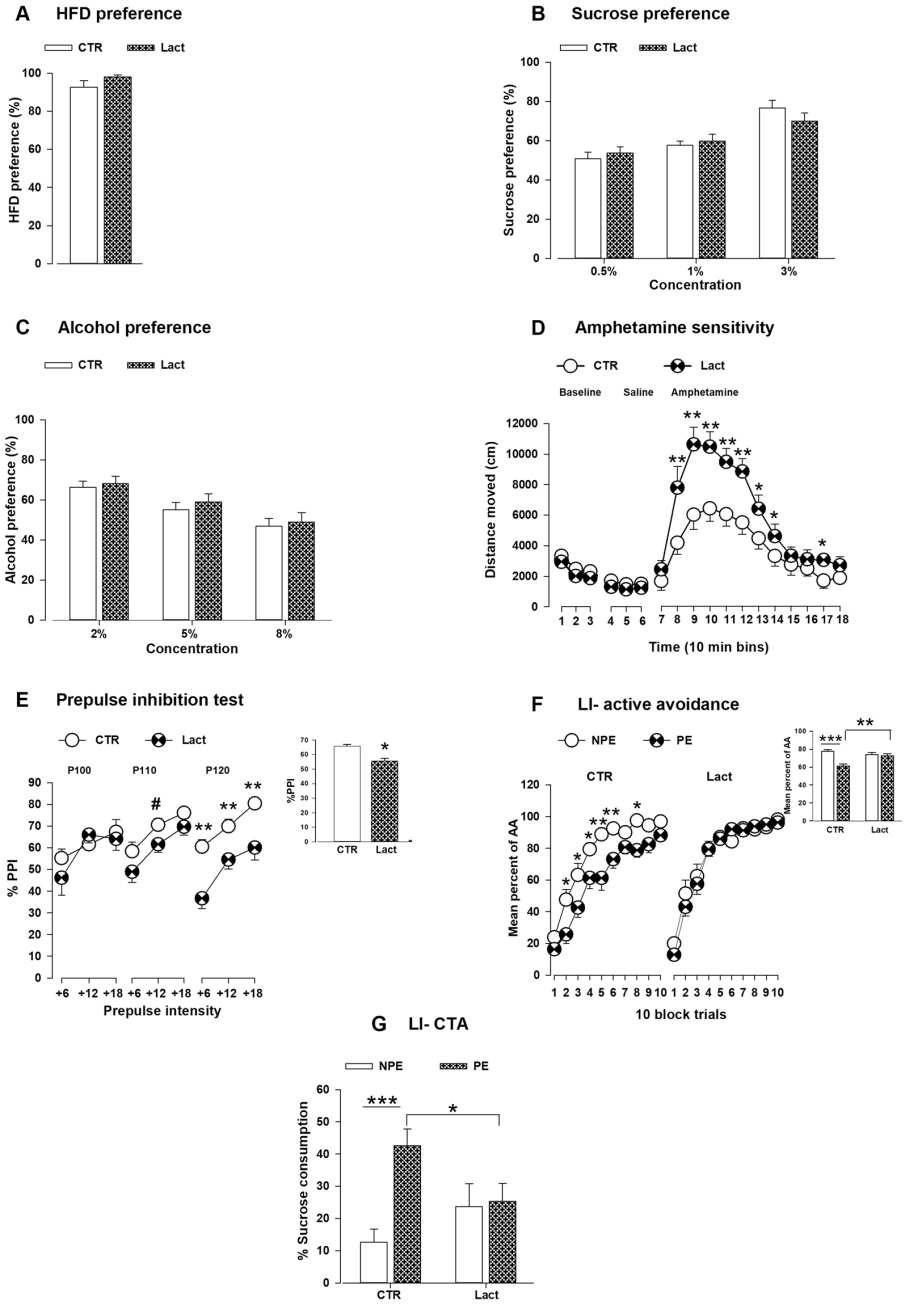
Effects of maternal HFD exposure during lactation on the behavior of the offspring. (**A**) HFD preference test: the bar graph shows the mean HFD preference in the lactation and CTR group. N = (6 m, 6f) per group**. (B)** Sucrose preference test: the bar graph shows the mean sucrose preference in different concentrations (0.5%, 1% and 3%). N = (10 m, 10f) per group. **(C)** Alcohol preference test: the bar plot displays the mean alcohol preference at three different concentrations (2%, 5% and 8%). N = (12 m, 12f) per group**. (D)** Amphetamine sensitivity: the line plots show the distance travelled in successive 10 min bins following baseline, saline and a systemic amphetamine injection in the open field paradigm. N = (5 m, 5f) per group. **(E)** %Prepulse inhibition of the acoustic startle reflex: the line graph shows the percent PPI as a function of different pulses (100, 110 and 120 dB) and the corresponding prepulse intensities (+6, +12 and +18 DB above the background 65 dB). The bar plot depicts the comparison of the mean %PPI between the groups. N = (10 m, 10f) per group. **(F)** LI-active avoidance: the line plot shows the percentage of avoidance responses made during 100-trial test sessions in the conditioned two-way active avoidance paradigm. The bar graph shows the average of the total percentage avoidance responses for the 100-trial tests. N = (10 m, 10f) per group and condition. **(G)** LI-conditioned taste aversion (CTA): the bar graph displays the percentage of sucrose consumption in the pre-exposed and non pre-exposed mice from both lactation and CTR group. N = (10 m, 10f) per group and condition. All values are mean± s.e.m. *P < 0.05; **P < 0.001; ***P < 0.0001. f, female; m, male. CTR = control; Lact = lactation; HFD = high fat diet; PE = pre-exposure; NPE = non pre-exposure.

In the HFD preference test, no difference in HFD preference was detected between the lactation and CTR offspring (*F*_1,20_ = 1.79; *P* = 0.19; Fig. 4A). In the sucrose preference test, offspring from both groups preferred the sucrose solution in the higher concentrations to water (main effect of solution, *F*_1,34_ = 21.59; *P* < 0.0001 and concentrations, *F*_2,68_ = 38.05; *P* < 0.0001). No difference was detected in sucrose preference between the groups (Fig. 4B). Further, in the alcohol preference test, offspring from both groups drank more alcohol in the lowest concentration than water (main effect of concentration, *F*_2, 84_ = 36.62; *P* < 0.0001) (Fig. 4C). As depicted in Fig. 4D, no difference in spontaneous locomotor activity at baseline and following injection of saline was detected between the offspring groups. Lact offspring showed significantly higher locomotor activity in response to amphetamine compared to CTR offspring as was supported by a significant main effect of group (*F*_1,19_ = 9.30; *P* < 0.008) and a significant interaction of group × 10-min bins (*F*_11,209_ = 4.67; *P* < 0.0001). In the PPI test, %PPI was increased with the increased prepulse intensities in both groups (main effect of prepulse intensities, *F*_2,70_ = 51.74; *P* < 0.0001) **(**Fig. 4E**)**. Disruption of % PPI was observed in Lact offspring as they showed an overall reduction of % PPI across all pulse-prepulse intensities compared to CTR offspring (*F*_1,35_ = 6.91; *P* < 0.02). % PPI was significantly reduced at the highest pulse intensity (120 dB) which was supported by an interaction of group × pulse intensities (*F*_2,70_ = 3.23; *P* < 0.05). *Post hoc* analyses revealed that % PPI deficit in the lactation group at pulse 120 dB was evident across all three prepulse intensities (+6 dB, +12 dB and +18 dB) (6 dB: *P* < 0.0003; 12 dB: *P* < 0.009; 18 dB: *P* < 0.002). We further assessed the associative learning ability by testing LI effect in the active avoidance and conditioned taste aversion (CTA) paradigms. In the LI-active avoidance experiment, acquisition of avoidance response was improved in all offspring from both conditions over the 10-blocks which was supported by a main effect of block (*F*_9,504_ = 185.11; *P* < 0.0001) **(**Fig. 4F**)**. A reduction in avoidance responses in the pre-exposed (PE) compared to the non pre-exposed (NPE) groups constituted the LI phenomenon. In general, NPE mice showed increased avoidance response learning compared to the PE group (main effect of pre-exposure, *F*_1,56_ = 8.09; *P* < 0.007). A significant interaction of group and pre-exposure was revealed (*F*_1,56_ = 10.04; *P* < 0.004). Subsequent *post hoc* analysis depicted reduced avoidance responses in the PE group compared to the NPE group in CTR offspring (*P* < 0.0001). In contrast, no difference was observed in avoidance responses between the PE and NPE groups in the Lact offspring. Further, the PE-lactation offspring group showed significant higher avoidance responses compared to PE-CTR offspring (*P* < 0.0008). Together, these findings indicated that a disruption of LI was evident in Lact offspring. In the LI-CTA paradigm, LI was indexed by the relative increase of sucrose consumption in the PE compared to the NPE animals. ANOVA revealed a significant main effect of pre-exposure (*F*_1,40_ = 7.99; *P* < 0.008) that demonstrated the overall existence of LI. In addition, a significant group × pre-exposure effect was observed (*F*_1,40_ = 6.44; *P* < 0.02) **(**Fig. 4G**)**. Subsequent *post hoc* comparisons indicated that the LI effect was evident in the CTR group (*P* < 0.0002), i.e., the decreased suppression of sucrose intake in the PE compared to NPE CTR mice. Such a difference was not evident in the lactation group. Moreover, the PE-lactation group showed enhanced suppression of sucrose intake compared to the PE-CTR group (*P* < 0.04). These observations suggested that the Lact offspring displayed impaired LI in the CTA paradigm.

### Neurochemical alterations in the HFD exposed offspring during critical periods of development

**Figure 5:**
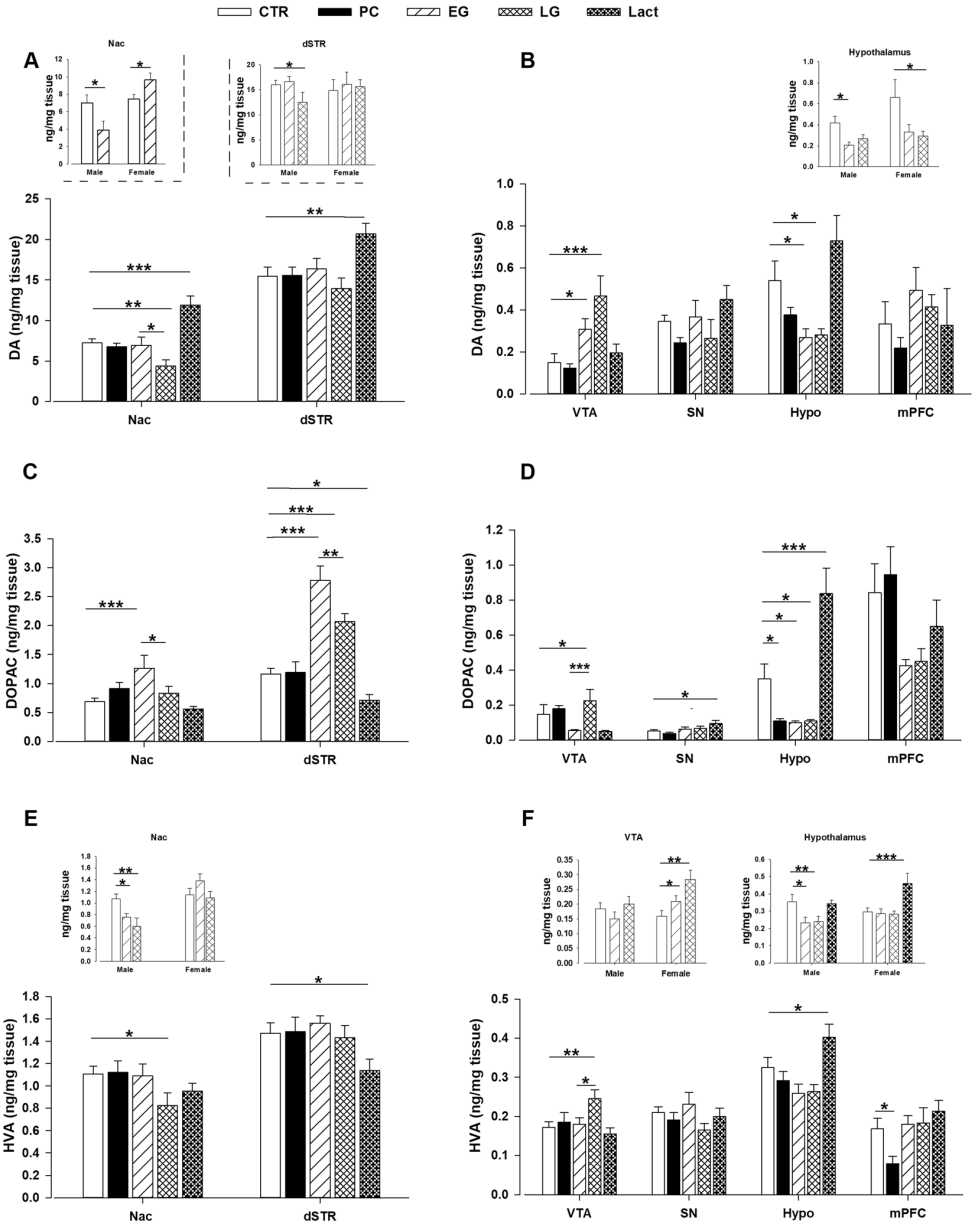
Levels of dopamine and its metabolites in the offspring exposed to maternal HFD during critical periods of development. Monoamines were measured in the Nac, dSTR, VTA, SN, Hypo and mPFC by postmortem HPLC and expressed as ng/mg tissue weight. **(A,B)** DA contents in the PC, Sem 1, Sem 2 and Lact offspring compared to the CTR. The inlets show the DA level in the Nac, and hypothalamus of male and female offspring born to early and late gestation HFD exposed dams. **(C)** and **(D)** DOPAC levels in the PC, Sem 1, Sem 2 and Lact offspring compared to the CTR. **(E,F)** HVA content in the PC, Sem 1, Sem 2 and Lact offspring compared to the CTR. The inlets showing the sex dependent alteration of HVA levels in the Nac, VTA and Hypothalamus of Sem 1, Sem 2 and lactation offspring. N = (8 m, 8f) per group. All values are mean ± s.e.m. *P < 0.05; **P < 0.001; ***P < 0.0001. f, female; m, male. Lact = lactation; CTR = control; HFD = high-fat diet; Nac = nucleus accumbens; dSTR = dorsal striatum; VTA = ventral tegmental area; Sn = substantia nigra; Hypo = hypothalamus; mPFC = medial prefrontal cortex; PND = postnatal day.

We next measured and compared the levels of DA and its metabolites (DOPAC and HVA) in each brain region (Nac, dSTR, VTA, SN, Hypo and mPFC) from the different offspring groups. We observed that MHFD exposure during different stages of development altered the levels of DA in the Nac in a group and a sex specific manner as indicated by the main effect of group (*F*_4,68_ = 11.24; *P* < 0.0001) and sex (*F*_1,68_ = 16.00; *P* < 0.0002) as well as a significant interaction of group × sex (*F*_4,68_ = 4.09; *P* < 0.006; Fig. 5A). Subsequent *post hoc* analysis yielded that both male and female Lact offspring showed significantly higher DA levels in the Nac (*P* < 0.0001) compared to CTR offspring **(**Fig. 5A**)**. EG female offspring had higher DA level (*P* < 0.03) while EG male offspring had lower DA level (*P* < 0.05) in the Nac compared to their controls. Both male and female LG offspring showed lower dopamine levels in the Nac compared to control (*P* < 0.006) as well as EG offspring (*P* < 0.03) **(**Fig. 5A**)**. In the dSTR, the levels of DA were altered among different groups (main effect of group, *F*_4,74_ = 3.34; *P* < 0.01; Fig. 5A). Both male and female Lact offspring showed increased DA levels compared to control offspring (*P* < 0.006). In contrast, only LG male offspring had lower DA levels compared to CTR (*P* < 0.05) as well as EG (*P* < 0.04) offspring. In the VTA, ANOVA revealed a significant main effect of group (*F*_4,71_ = 6.14; *P* < 0.0004) and sex (*F*_1,71_ = 4.86; *P* < 0.04), indicating that MHFD exposure at different time periods altered the levels of dopamine in this region. *Post hoc* analysis showed that both EG (*P* < 0.04) and LG (*P* < 0.0001) offspring had higher DA levels in the VTA compared to their controls **(**Fig. 5B**)**. A significant group and sex interaction (*F*_4,76_ = 2.53; *P* < 0.05) was also detected in hypothalamic DA levels where EG male (*P* < 0.02) and LG female (*P* < 0.05) offspring showed lower DA levels compared to control offspring **(**Fig. 5B**)**. No difference in DA levels was detected between PC and CTR offspring in all six-brain regions.

The levels of DOPAC were also influenced by MHFD exposure at critical periods depending on different brain regions. In the Nac, ANOVA revealed a significant main effect of group (*F*_4,68_ = 5.69; *P* < 0.0006), sex (*F*_4,68_ = 15.16; *P* < 0.0003) and a group × sex interaction (*F*_4,68_ = 6.50; *P* < 0.0003). Subsequent *post hoc* analysis revealed a significantly higher DOPAC level in the EG offspring as compared to the CTR (*P* < 0.0006) and LG (*P* < 0.03) offspring **(**Fig. 5C**)**. In the dSTR, significantly higher DOPAC levels were detected in the EG (*P* < 0.0001) and LG (*P* < 0.0001) offspring but lower DOPAC levels in the Lact (*P* < 0.05) offspring compared to the CTR **(**Fig. 5C**)**. Further, a higher DOPAC levels were observed in the VTA of LG offspring compared to CTR (*P* < 0.003) and EG (*P* < 0.0004) offspring **(**Fig. 5D**)**. In the hypothalamus, Lact offspring showed increased DOPAC levels (*P* < 0.0001) whereas the rest of the groups showed reduced DOPAC levels (*P* < 0.03) compared to the CTR **(**Fig. 5D**)**.

The levels of HVA were altered in the Nac of different offspring groups as was supported by a significant group × sex interaction (*F*_4,68_ = 2.55; *P* < 0.05). *Post hoc* analysis depicted that only male offspring from EG (*P* < 0.03) and LG (*P* < 0.002) groups showed lower HVA levels compared to the CTR **(**Fig. 5E**)**. The levels of HVA were also lower in the dSTR of Lact (*P* < 0.03) **(**Fig. 5E**)** and the mPFC of PC offspring (P < 0.03) relative to the CTR **(**Fig. 5F**)**. In contrast, higher levels of HVA were observed in the VTA of LG female offspring compared to CTR (*P* < 0.005) as well as EG (*P* < 0.05) offspring **(**Fig. 5F**)**. In the hypothalamus, EG male offspring (*P* < 0.04) and LG (*P* < 0.05) showed lower HVA levels whereas Lact female offspring showed increased HVA levels (*P* < 0.0007) compared to CTR **(**Fig. 5F**)**.

## Discussion

In the present study, we observe that time specific MHFD exposure is associated with profound metabolic alterations, enhanced addictive-like and schizophrenia-relevant behaviors in the offspring later in life. Postmortem neurochemical analysis further show that the metabolic and behavioral abnormalities are accompanied with sustained alterations in DA levels in the reward related brain regions. The main findings are summarized in Table 1.

**Table 1:** The long-term effects of maternal HFD exposure during preconception, early and late gestation and lactation in different aspects of behavior and metabolism in adult offspring.

Assessments	Preconception	Early Gestation	Late Gestation	Lactation
Body weight	=	=	=	↑
Fat mass ratio	ND	ND	ND	↑
Insulin insensitivity	ND	ND	ND	↑
Lipid profile	ND	ND	ND	↑
HFD preference	=	=	=	=
Sucrose preference	=	=	=	=
Alcohol preference	=	=	↑	=
AMPH sensitivity	=	↑	↑	↑
Prepulse inhibition	=	=	=	↓
LI-active avoidance	ND	ND	ND	↓
LI-conditioned taste aversion	ND	ND	ND	↓

(=) = no difference between treatment and control (CTR) groups; (↑) = increased relative to CTR; (↓) = decreased relative to CTR; ND = not done, further experiments were not conducted.

We observe that only Lact offspring show significant weight gain from weaning throughout adulthood compared to CTR offspring. Consistently, Lact offspring show higher fat content, larger lipid droplets in the visceral fat depot and impaired insulin sensitivity which might further potentiate the development of hypercholesterolemia and hypertriglyceridemia in this offspring group^33^. Notably, such metabolic abnormalities are apparent in both male and female offspring. Our findings are in line with previous studies showing that MHFD feeding during lactation have the strongest effects on the development of obesogenic and or diabetogenic phenotypes in adult offspring^28,34,35^. These metabolic effects may be due to changes in mammary gland morphology or the specific nutritional or hormonal composition of the dams’ milk^28,36^. The metabolic abnormalities, at least in part were shown to be via impaired neuronal innervation from the arcuate nucleus of the hypothalamus to intrahypothalamic target areas^28^. Conversely, we observe that 3 weeks preconception HFD exposure does not affect the metabolic health of the offspring. Opposed to our findings, an earlier study report that 3–4 weeks preconception MHFD exposure increases weight gain, adiposity and hepatic lipid droplet size in the adult offspring^26^. The key difference is that our MHFD model does not induce an obesogenic phenotype in HFD dams^22^. Moreover, species differences as well as fat and calorie content of the diet needs to be considered. Similarly, no body mass difference is observed following HFD exposure during early or late gestation compared to control offspring. One possible explanation could be that the duration of exposure is too short (only 11 days) to induce metabolic abnormalities. Our findings corroborate earlier studies showing that HFD exposure during gestation alone or defined periods of gestation induces minor or no alterations of plasma glucose, insulin and leptin levels with no changes in body mass or adiposity in the offspring^27,37^. In accordance with previous studies^38,39^, our data suggest that the programming effect of maternal obesity or overnutrition might be initiated from the onset of pregnancy, however, the crucial period is the dietary pattern during the lactation phase. Maternal dietary pattern during breastfeeding and its influence on weight gain and the poor feeding habits in children has also been reported in several human studies^40^. Human studies demonstrate differences in weight gain between breastfed infants and formula fed or mixed fed infants^41–43^. Moreover, postpartum weight gain in mothers can be a predictor of future metabolic risks in children^44^. Nutritional diet during breastfeeding are critical for the child health since the flavours of breastmilk experienced by some infants may be influenced by the diet of the mother^45^. A direct comparison of our findings to human subjects warrants caution, based on the difference of the developmental trajectory between rodents and humans^46^. Nevertheless, our data support the human observation and emphasizes the importance of proper diet selection during breastfeeding to lessen the risk of obesity or overweight in future generations.

One of the major findings in the current study is the clear dissociation of behavioral phenotypes in the offspring exposed to MHFD at different time windows. Neither the hedonic response to natural rewards nor the response to drugs of abuse is altered following preconception MHFD exposure. Similarly, 2 months preconception HFD exposure does not alter motivational behaviors tested in progressive ratio task and sucrose preference tests in the rat offspring^47^. MHFD during early and late gestation induces increased sensitivity to amphetamine whereas increased alcohol consumption is observed only following late gestational HFD exposure. Hedonic response to natural rewards does not differ between the offspring groups. We previously showed that long term MHFD exposure (3 weeks preconception and 6 weeks during gestation and lactation) induces increased palatable food preference (HFD and sucrose) as well as increased sensitivity to drugs of abuse (alcohol, amphetamine and cocaine)^22^. Taken together, these data imply that the developmental time period during late gestation might be more vulnerable for increased susceptibility to addictive-like behaviors following a HFD insult.

On the other hand, we have found that lactational MHFD exposure induces schizophrenia-like behaviors in the offspring, for instance, PPI impairment and disruption of LI. In addition, Lact offspring show increased amphetamine-induced locomotor activity. Disruption of PPI, which refers to sensory motor gating deficits, and LI disruption, which reflects attention deficits, have been commonly reported in schizophrenic patients with marked positive symptoms^48,49^. Human PET studies show that a systemic low dose amphetamine induces psychotic symptoms in normal subjects and exacerbates positive symptoms in schizophrenic patients by stimulating striatal DA release^50,51^. Such findings in human studies are also documented in rodent models of schizophrenia^52,53^. Based on these findings, our data suggest that MHFD exposure during lactation predisposes the offspring to develop positive symptoms of schizophrenia. Recent research from human case control and cohort studies provides a positive correlation between pre-pregnancy BMI or gestational weight gain and increased predisposition towards schizophrenia in the adult offspring^54,55^. However, no animal studies have been conducted so far to translate such observations. To the best of our knowledge, the present study is the first to show in a mouse model that MHFD exposure exclusively during lactation is sufficient to induce schizophrenia-related abnormalities in the offspring later in life.

Another key observation in the present study is that concomitant to schizophrenic traits Lact offspring develop the metabolic syndrome. Schizophrenia is linked to multiple metabolic abnormalities including obesity, type 2 diabetes and cardiovascular diseases^56,57^. The presence of such metabolic abnormalities is not solely an effect of antipsychotic drug treatment. Accumulating evidence report the occurrence of metabolic dysfunction in psychosis-prone subjects as well as in drug naïve schizophrenic patients, which might be linked to environmental insults during development^58,59^. Indeed, Pacheco-Lopez, *et al*.^60^ show in a mouse model of prenatal infection that adult male offspring develop a schizophrenia-like phenotype that is accompanied by metabolic syndrome related phenotypes. In contrast, Zieba and co-workers reported that maternal obesity (6 weeks prior to mating and during gestation and lactation) increased offspring’s’ body weight however improved sensorimotor gating compared to offspring of control mothers^20^.

The neural circuitry responsible for the pathophysiology of obesity and neuropsychiatric disorders is highly complex and involves multiple neurotransmitter systems. However, convergence evidence suggests a crucial role of the mesolimbic dopaminergic system in the pathogenesis of addiction^61^, schizophrenia^62^ and obesity^63^. We therefore hypothesize that the behavioral and metabolic abnormalities in offspring exposed to MHFD during different time windows might be partly explained by the neurochemical changes in the mesolimbic dopaminergic system. Although, we do not exclude that other neurotransmitter systems are involved. Preconception MHFD exposure does not induce any changes in DA levels in the offspring. However, significant changes in DA and its metabolites are detected in the early and late gestation HFD exposed offspring. In EG offspring, a dissociation between female and male offspring emerged exclusively in the Nac region where lower DA and HVA levels were detected in male offspring and higher DA levels in the female offspring compared to their controls. In the LG group, both male and female offspring show reduced DA levels in the Nac whereas only male offspring show lower DA levels in the dSTR compared to the other groups. In addition, higher DA and its metabolites levels are detected in the VTA of both gestational HFD exposed offspring, suggesting a compensatory mechanism in this region to overcome altered DA levels in the striatum. Taken together, our data indicate a hypodopaminergic state in the striatal regions, which is more prominent in late gestation MHFD exposed offspring. Similar alterations in striatal DA levels are reported in chronic methamphetamine users^64^ as well as rodent models of addiction following withdrawal from cocaine^65^, amphetamine^66^ and ethanol^67^. Such an altered mesolimbic system is also observed in our previous model of 9 weeks MHFD^22^. The current findings extend our previous observations that short-term MHFD exposure during late gestation is sufficient to induce a hypodopaminergic state, which could potentiate the preference for alcohol and increased sensitivity to drugs of abuse in the offspring. On the other hand, both male and female Lact offspring show higher basal DA levels in the Nac and dSTR compared to CTR offspring. The levels of DOPAC and HVA are significantly low in the dSTR of this offspring group. Besides other significant changes, the higher DA levels in the Nac and dSTR together with lower DOPAC and HVA levels indicate a baseline hyperdopaminergic state in the striatal regions which might play a crucial role in the emergence of positive symptoms of schizophrenia in this offspring group. This interpretation is in line with studies showing that elevated baseline striatal DA levels and increased presynaptic DA release in the Nac and dSTR following a challenge injection of amphetamine is correlated with positive symptoms of schizophrenia in drug naïve schizophrenic patients^62,68^ as well as in other neurodevelopmental rodent models of schizophrenia^69^. Such hyperdopaminergic state might also explain the concomitant presence of the metabolic syndrome in this offspring group. Abnormalities in insulin signaling pathway and its components for example, PIP2/PKC/PLC activity are strongly associated with the hyperdopaminergic theory of schizophrenia. It is postulated that elevated DA levels observed in schizophrenic patients with positive symptoms could induce central insulin resistance via the downregulation of PKC/PLC/AKT/GSK and glucose transporter activity, leading to a hyperglycemic state. Hyperglycemia might further precipitate the positive symptoms of schizophrenia by increasing the activity of DA in limbic brain regions^70,71^. Further, central insulin resistance could potentiate the development of obesity and peripheral insulin resistance via the disruption of insulin signaling in the homeostatic and hedonic circuitry, alteration of hepatic lipogenesis and white adipose tissue metabolism^31,72^. In view of these effects, we could hypothesize that the hyperdopaminergic state observed in the Lact offspring could contribute to the development of central insulin resistance by disrupting several components of insulin signaling pathways, which will further exacerbate obesity and the metabolic syndrome.

One of the limitations of the current study is that the underlying mechanism responsible for the emergence of hyperdopaminergic and hypodopaminergic state of the mesolimbic system following MHFD exposure remains essentially unexplored. From a developmental perspective, the midbrain dopaminergic neurons development starts during G10-G13 in the mouse embryo where mainly the DA neurons proliferate and differentiate from the precursor cells. The migration, innervation in target regions and synapse formation take place in the second half of gestation^32^. Whereas structural and functional maturation including increased DA receptor concentrations in the ventral and dorsal striatum occurs in the first 3 weeks of post-natal life^73^. One possible explanation would be that the MHFD consumption initiate at different stages of development especially late gestation and lactation could directly affect the structural and functional maturation of dopaminergic neurons, which lead to altered DA levels later in life. Moreover, increased oxidative stress and pro-inflammatory cytokines because of MHFD exposure during gestation and lactation might also negatively affect the development of dopaminergic neurons^74,75^.

In conclusion, the present study for the first time provides clear insight in to the relative contribution of MHFD exposure at different stages of preconception, gestation and lactation to induce distinct brain pathology and phenotypes related to metabolic and mental health related disorders. The major findings of our study are: (1) short-term preconception HFD exposure does not affect offsprings’ brain, behavior and physiology. (2) Late gestation HFD exposure induces most features of addictive-like behaviors that is further substantiated by the presence of lower striatal DA levels. (3) MHFD exposure during lactation leads to increased striatal DA levels, which may precipitate the positive symptoms of schizophrenia-like symptoms and the obesogenic trait in the offspring. These observations urge further investigations to identify the underlying mechanisms for such time specific MHFD insult and to provide novel targets for early therapeutic interventions to be able to reverse the epidemic of obesity in women at childbearing age and in turn alleviate health consequences in the offspring.

## Materials and Methods

### Animals

Male and female C57BL/6N mice (Charles River, Germany) were housed four per cage (same-sex) under standard conditions at 22 °C on a 12:12 h reversed light cycle (lights off 7 am–7 pm) and had ad libitum access to food and water. All mouse experiments described in this study were carried out in strict accordance with the recommendations in the Animal Welfare Ordinance (TSchV 455.1) of the Swiss Federal Food Safety and Veterinary Office. It was approved by the Zurich Cantonal Veterinary Office, Switzerland

### Feeding and breeding regime

Female mice were assigned to receive either standard laboratory chow (Kliba-Nafag 3430, Klibamühlen or HFD (60% energy from fat, Kliba- Nafag 2127, Kaiseraugst, Switzerland). The major nutrients of the chow diet are 18.5% crude protein, dry matter 88%, crude fat 4.5%, 54.2% nitrogen free extract (NFE). The total energy content is 3.2 kcal/g, protein 21%, carbohydrates 39.8% and fat 5% (% of total kcals). The major nutrients of HFD: 23.9% crude protein, dry matter 92%, crude fat 35%, 23.2% NFE. It contains 26% protein, 38% fat and 1% carbohydrates with total energy 22 kJ/g or 5.25 kcal/g^76,77^.

**Figure 6:**
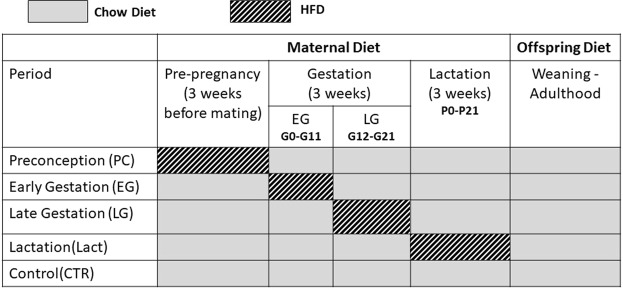
Experimental design illustrating the breeding scheme and dietary pattern. Control (CTR) dams are maintained on standard laboratory chow diet during preconception, gestation and lactation. In case of high-fat diet (HFD) exposure, dams were exposed to HFD during specific periods of gestation and lactation. In the preconception (PC) group, mothers are fed HFD 3 weeks prior to conception. In the early (EG) and late (LG) gestation groups, dams are exposed to HFD during GD0–11 or during GD 12-21, respectively. In the lactation (Lact) group, mothers are fed HFD only during the lactation period. Offspring from all groups are maintained on standard chow diet from weaning and throughout adulthood. GD = gestation day; P = Postnatal day.

Females were divided into four groups: (a) preconception (*PC*): female dams received HFD only 3 weeks prior to mating and switched to normal chow for the rest of the period of gestation and lactation; (b) EG: female mice were exposed to HFD from gestational day G0 to G11; (c) LG: pregnant dams received HFD only from G12 to G21; (d) lactation *(Lact)*: dams received HFD only during lactation [from birth of the pups until postnatal day (PND) 21]. The control (CTR) dams received chow during this 9 weeks period. The groups were summarized in Fig. 6. The male breeders were exposed to chow and removed from the mating cage as soon as the copulation plug was confirmed.

We evaluated before different metabolic traits of the dams that were exposed to either HFD or chow at preconception, early and late gestation and lactation in a separate cohort of mice^22^. No difference in body mass, fat mass distribution, blood glucose and insulin as well as plasma lipid levels were detected between dams that were exposed to HFD during different time lengths or chow (Peleg-Raibstein *et al*., 2016; Supplementary Figs. 1–4^22^).

The pups were weaned at PND 21 and were housed four littermates of same sex per cage. No difference in litter size was detected between the different treatment groups (Supplementary Information, Table 1). Offspring from all groups were exposed to chow and water ad libitum throughout their life. All the experiments were conducted when the mice reached adulthood (PND 70–110). To avoid sex^78^ and litter^79^ effect, maximum one male and one female offspring from each litter of every treatment groups were allocated in each behavioral, metabolic and neurochemical experiment. Ten offspring from both sexes from 10 different litters were used for HFD, sucrose and alcohol preference test. The HFD, sucrose, and alcohol preference tests were conducted chronologically with the same set of animals with a wash time period of at least 7 days in between tests. For the amphetamine sensitivity test, an independent naïve cohort of offspring was allocated from each experimental group. PPI, LI-active avoidance and LI-CTA were conducted in an independent cohort of offspring from each experimental group. Behaviorally naïve animals were chosen from each sex and group for the metabolic and neurochemical analysis. For all the experiments, five independent cohort of offspring were employed. A detailed description of the animal numbers used in each experiment can be found in the Supplementary Information as Supplementary Tables 9–11. All behavioral experiments were carried out during the dark phase (between 9 am to 5 pm) under red light or under dim light illumination.

### Behavioral experiments

#### High fat diet preference test

In the HFD preference test, mice were single caged in type II cages without bedding for 3 hours during the test period. One day prior to the test, the mice were habituated to the altered home cage condition to reduce stress during the test. Animal body mass was measured daily prior to testing. During the test, mice were exposed to both laboratory chow diet and HFD for 3 hours. The test was conducted for four consecutive days (same time every day). During the test, mice had ad libitum access to water. The weight of the foods was measured before and after the test. For data analysis, an average of the last 3 days was taken, and HFD preference was calculated as percentage of HFD intake in relation to total food intake^23^.

#### Sucrose preference test

The protocol for sucrose preference test was validated in our laboratory before and described in detail^22^. On day 1 and 2, two bottles contained water and mice were habituated to drink from both bottles (habituation phase). From day 3, one of the drinking bottles was filled with sucrose solution and the other with water. Three different sucrose concentrations were tested and each concentration was assessed for a 3-day period. Consumption of water, sucrose and body mass were measured every day. The sucrose preference was calculated as percentage of sucrose consumption in relation to total fluid intake^23^.

#### Alcohol preference test

The same protocol was used as for the sucrose preference test [see above^22^,]. The alcohol preference was calculated as percentage of alcohol intake in relation to total fluid intake.

#### Amphetamine-induced locomotor activity

The locomotor activity after a systemic amphetamine injection was tested in an open field arena as described elsewhere^22^. Animals were placed in the open field arena and basal locomotor activity was recorded for 30 min. The mice were then injected with (intraperitoneal; i.p) 0.9% NaCl solution and after 30 min of locomotor assessment, mice were i.p injected with d-amphetamine sulfate (2.5 mg/kg). Activity was monitored for 2 hours. Data were indexed by the distance travelled in the entire arena and summed for each successive 10 min bins.

#### Percentage prepulse inhibition (%PPI)

Sensory motor gating was examined using the prepulse inhibition (PPI) of the acoustic startle reflex paradigm according to a protocol established before^80^. The test was conducted in four ventilated startle chambers (SR-LAB, San Diego Instruments, San Diego, CA, USA). The motion inside the tube was recorded by a piezoelectric accelerometer below the frame. The amplitude of the whole body startle response was defined as the average of 100 ms accelerometer readings collected from the pulse onset. At the beginning of the session, mice were placed in the startle chamber and acclimatized for 2 min with a 65 dB background noise level. After this habituation period, 10 blocks of 16 discrete trials were presented in a pseudorandom manner to assess PPI. Each block consisted of four different trial types: pulse alone (one trial for each pulse intensity: 100, 110 and 120 dB), prepulse-alone trials (one trial for each prepulse intensity including +6, +12 and +18 dB above background), prepulse-pulse trials (one trial for each prepulse-pulse combination) and a single no stimulus trial (only the background noise). %PPI was indexed by the following formula: [(startle during pulse-startle during prepulse-pulse)/startle during pulse] × 100.

#### Latent inhibition in two-way active avoidance paradigm

Latent inhibition was tested only in the MHFD lactation groups since this groups showed schizophrenia-related behavioral abnormalities^81^ compared to the other MHFD offspring groups. We assessed latent inhibition (LI) as a test of selective attention in a two-way active avoidance paradigm as described elsewhere^82^. LI refers to a phenomenon where repeated non-reinforced pre-exposure to a conditioned stimulus (CS) impairs the subsequent development of a conditioned response (CR) following explicit pairings of the same conditioned stimulus (CS) and an unconditioned stimulus (US)^83^. The apparatus was consisted of four identical two-way shuttle boxes (model H10-11 M-SC; Coulbourn Instruments). An electric shock of 0.3 mA was the US and the CS was an 83 dB white noise. The experiment included two phases: pre-exposure and conditioning phase. The animals from both treatment groups were divided into two conditions: pre-exposure (PE) and non-pre-exposure (NPE). In the pre-exposure phase, PE animals were placed in the shuttle chambers and received 50 presentation of 5 s white noise CS presented at a random interval of 40 ± 15 s. The NPE animals spent equivalent period in the chambers without any stimulus presentation. During the conditioning phase, the animals were placed back to the same chambers and were presented with 50 conditioned avoidance trials with an interval of 40 ± 15 s. A trial started with the onset of the CS. If the animal shuttled within 5 s of CS onset, the CS was terminated and the animal avoided the electric shock on that trial (avoidance response). Avoidance failure immediately induced an electric foot shock presented in coincidence to the CS. This could persist for a period of 2 s but could be ended by a shuttle response during this period (escape response). To index the conditioned avoidance learning, the mean latent response across consecutive 10-trail blocks was calculated for analysis.

#### Latent inhibition in a conditioned taste aversion (CTA) paradigm

LI was also assessed in the CTA paradigm as previously described in detail elsewhere^84^. The testing was conducted daily between 10 am and 4 pm during the dark phase. The animals were gradually acclimatized to a water-deprivation schedule over a 6-day period until they were kept on 23 hours water deprivation, which was maintained throughout the experiment. Body mass was monitored daily. The consumption was determined by calculating the difference in the weight of the drinking tube before and after the test session. The experiment consisted of 4 phases: baseline, pre-exposure, conditioning and the test.

Baseline: On days 1–4, water was provided in both tubes for two 30 min sessions at 12 pm and 4 pm. The position of the tubes was changed between sessions to minimize side preference. At the end of day 4, the animals were subdivided into two groups: pre-exposed (PE) and non-pre-exposed (NPE), with their baseline drinking counterbalanced.

Pre-exposure: On day 5, PE and NPE animals were given access to 10% sucrose and water respectively in both tubes in the first drinking session (12 pm–12.30 pm). All animals received water in the second drinking session.

Conditioning: On day 6, all animals received 10% sucrose in both tubes for 30 min in the first drinking session, followed by an immediate i.p injection of 0.25 M lithium chloride (LiCl) solution at a dose of 2% body mass. LiCl induced gastric nausea and served as the US. All animals received water in the second drinking session.

Test: On day 7, the mice were given free access to two drinking tubes, one contained 10% sucrose and the other was filled with water in the first drinking session. Percentage of sucrose intake was calculated by the following formula = [sucrose consumed in ml/ (sucrose + water consumed in ml]*100. Reduced aversion in the PE animals compared to the NPE animals constituted LI.

### Metabolic phenotype

#### Body mass

Body mass of each offspring were measured weekly from PND 21 up to PND 77 with an electronic scale (Mettler PM 2000).

#### Insulin tolerance test

The animals were food deprived for 6 hours before the experiment. After measuring the fasted blood glucose levels, insulin (Actrapid; Novo Nordisk A/S) was administered i.p at a dose of 0.75 IU insulin/kg body mass in males and 0.6 IU insulin/kg body mass in females. Glycaemia was measured 30, 60, 90 and 120 minutes after the injection. Glucose level was measured in fresh tail blood using Accu-Chek Aviva device (Roche).

#### Computed tomography (CT)

The distribution of body fat was measured using a computed tomography (CT) X-ray scanner (La Theta LCT-100; Aloka Inc, Japan) as described before^22^. The CT scanner measures the volumes of adipose tissue, bone, air and the rest based on the differences in X-ray density and distinguishes between intra-abdominal and subcutaneous adipose tissue. Following sedation with 4–5% isoflurane, mice were laid in supine position in the mouse holder where the gas anesthesia (1.5–2.5% isoflurane) was maintained during scanning. First, the scan area was set based on sagittal pre-scan and fixed anatomical landmarks. A whole body scan excluding the head and tail was performed at a rate of 4.5 s/image for 5 min. The images were analyzed using the La Theta 2.10 software.

#### Plasma parameters insulin and lipid concentrations

For the collection of plasma samples, mice were food deprived for 6 hours prior to dissection. The blood was collected by cardiac puncture in an aliquot containing 5 ul EDTA (0.6 M) and centrifuged on 1000 × g for 10 min. To determine the plasma insulin level commercially available enzyme linked immunosorbent assay kit (ELISA; Crystal Chem Inc, USA) was used. The plasma levels of cholesterol (Chol; Roche, Switzerland), triglycerides (TG; Hitachi, Switzerland) and non-esterified free fatty acids (NEFA; Wako, Germany) were measured by enzymatic colorimetric assays.

#### Lipid droplet size measurement

Adult mice were euthanized by CO_2_. Adipose tissues were excised and stored in aliquots at −80 °C. The samples from visceral and subcutaneous adipose tissue were rapidly thawed using a water bath and then fixed in 4% paraformaldehyde (Sigma Aldrich) at pH 7.4. The tissue processing was performed according to the standard protocol in a tissue processor (STP 120; Microm). The samples were embedded in paraffin and cut into 5 um thin sections on a Hyrax M55 (Zeiss). Sections were de-paraffinized and stained with haematoxylin and eosin on a Varistain 24–4 (Thermo Scientific) before mounting and drying. Images were obtained using an Axioscope A.1 (Zeiss). The analysis of lipid droplet sizes was performed using CellProfiler (www.cellprofiler.org).

### Neurochemical analysis

#### Brain collection and tissue processing

Adult animals from all groups were euthanized by CO2 asphyxiation. At sacrifice, brains were rapidly extracted from the skull, placed on an ice chilled dissection plate covered with filter paper and cut with a sharp razor blade into 1 mm thick coronal sections. The discrete brain regions including the dorsal striatum (dSTR), nucleus accumbens (Nac), medial prefrontal cortex (mPFC), hypothalamus (Hypo), substantia nigra (SN) and ventral tegmental area (VTA) were isolated using a 1 mm micropunch. The brain regions were dissected according to the Mouse Brain Atlas in Stereotaxic Coordinates (Paxinos and Franklin, 2008) which were as follows: dSTR (bregma +1.34 to +0.14 mm), Nac (bregma +1.60 to +0.98 mm), mPFC (bregma +2.68 to + 1.94 mm), Hypo (bregma −1.34 to −2.06 mm), SN (bregma −2.80 to −3.64 mm), VTA (bregma −2.92 to −3.64 mm). Tissue punches from right and left hemisphere were combined, weighed and collected in an aliquot containing ice-cold 300 ul 0.4 M perchloric acid (HClO4). The samples were homogenized using a sonicator and centrifuged at 10,000 × g for 20 min at 4 °C. The supernatant was then collected and filtered through a 0.2 μm nylon filter (Spritzenfilter, Semadeni AG) to separate the insoluble residue. The supernatant was centrifuged again at 10,000 × g for 15 min at 4 °C to remove any residue and stored at −80 °C until further processing.

#### Post mortem high performance liquid chromatography (HPLC)

The levels of dopamine (DA) and its metabolites (dihydroxyphenylacetic acid, DOPAC; homovanillic acid, HVA) were measured by a HPLC system equipped with an electrochemical detector (Decade II; Antec, Leyden, The Netherlands) as previously described^85,86^. The sample (20 µl from each brain region) was injected via a refrigerated autoinjector (ASI-100, Dionex, CA, USA). The mobile phase was pumped through the system at a flow rate of 0.4 ml/min using an HPLC pump (P680, Dionex, CA, USA). Neurotransmitters were separated on a reversed-phase column (125 mm × 3 mm YMC column, Nucleosil 120–3C 18, YMC Europe GmbH, Germany). Data acquisition and calculations were performed using a chromatography workstation (Chromeleon, Dionex, Olten, Switzerland).

### Statistical analyses

Data were analyzed using the statistical software StatView (version 5.0). Analysis of variance (ANOVA) followed by post-hoc comparisons (Fisher’s least significant difference) or factorial ANOVA was employed whenever appropriate. For the weekly measurement of body mass, a 2 × 2 × 9 (maternal exposure × sex × weeks) for the preconception and the lactation groups and a 3 × 2 × 9 (maternal exposure × sex × weeks) repeated measures ANOVA for the gestation group were employed. To analyze the preference for HFD a 2 × 2 × 2 (maternal exposure × sex × food) for the preconception and the lactation groups and a 3 × 2 × 2 (maternal exposure × sex × food) ANOVA for the gestation group were used. For the sucrose and alcohol preference tests, a 2 × 2 × 2 × 3 (maternal exposure × sex × preference substance × substance concentrations) for the preconception and the lactation groups and a 3 × 2 × 2 × 3 (maternal exposure × sex × preference substance × substance concentrations) repeated-measures ANOVA for the gestation group were used. The baseline and saline phases prior to the amphetamine injection were subjected to a 2 × 2 × 3 (maternal exposure × sex × 10-min bins) for the preconception and the lactation groups and a 3 × 2 × 3 (maternal exposure × sex × 10-min bins) repeated-measures ANOVA for the gestation group. Amphetamine induced locomotor activity was subjected to a 2 × 2 × 12 (maternal exposure × sex × 10-min bins) for the preconception and the lactation groups and a 3 × 2 × 12 (maternal exposure × sex × 10-min bins) repeated-measures ANOVA for the gestation group. To analyze the % PPI, a 2 × 2 × 3 × 3 (maternal exposure × sex × pulse level × prepulse level) for the preconception and the lactation groups and a 3 × 2 × 3 × 3 (maternal exposure × sex × pulse level × prepulse level) repeated measures ANOVA for the gestation group were used. In the LI-active avoidance paradigm, a 2 × 2 × 2 × 10 (maternal exposure × sex × pre-exposure × 10-trials block) repeated measures ANOVA was employed. For the LI-CTA test, a 2 × 2 × 2 (maternal exposure × sex × pre-exposure) repeated-measures ANOVA was used. For the postmortem neurochemical analysis, the levels of dopamine, DOPAC and HVA were analyzed by factorial ANOVA (maternal exposure × sex) for each brain region. The statistical outcome was supported by a priori multiple comparisons, not a posteriori comparisons. This distinction is important in deciding the appropriate means to control for familywise Type-I error^87^. Statistical significance was set at P < 0.05. All data were presented as mean± standard error of mean (SEM). A summary of all statistical tests and outcomes can be found in the Supplementary Information, Tables 2–8.

## Supplementary information

Supplenentary Information
