# Supplementary material for: Maternal overnutrition during critical developmental periods leads to different health adversities in the offspring: relevance of obesity, addiction and schizophrenia
Source: Sci Rep. 2019 Nov 21;9:17322. doi: 10.1038/s41598-019-53652-x (PMC6872534; doi:10.1038/s41598-019-53652-x)
Supplement: Supplementary file 1 — Supplenentary Information [file 41598_2019_53652_MOESM1_ESM.docx]

**Maternal overnutrition during critical developmental periods leads to different health adversities in the offspring: relevance of obesity, addiction and schizophrenia**

**Supplementary Tables**

Authors

Gitalee Sarker, Kathrin Litwan, Rahel Kastli, *Daria Peleg-Raibstein

Affiliation

Department of Health Science and Technology, ETH Zurich, Schwerzenbach 8603, Switzerland.

Corresponding author:

*Dr. Daria Peleg-Raibstein

Department of Health Science and Technology

ETH Zurich, Schwerzenbach 8603

Switzerland

Tel. +41 44 655 73 50

E-Mail: daria-peleg@ethz.ch

**Supplementary Table 1**

| **Number of offspring** | **Preconception** | **Early Gestation** | **Late Gestation** | **Lactation** | **Control** |
| --- | --- | --- | --- | --- | --- |
| Male | 3.11 ± 0.34 | 4.84 ± 0.36 | 3.91 ± 0.15 | 4.41 ± 0.38 | 3.42 ± 0.14 |
| Female | 4.21 ± 0.55 | 3.99 ± 0.33 | 3.36 ± 0.15 | 4.33 ± 0.28 | 3.68 ± 0.13 |
| Total litter size | 7.33 ± 0.65 | 8.74 ± 0.30 | 7.27 ± 0.10 | 8.75 ± 0.30 | 7.1 ± 0.26 |

**Table S1:** Summary of the litter size in different maternal high-fat diet (MHFD) groups. No significant difference in male, female and total number of offspring per litter was observed between the control offspring and the offspring exposed to MHFD at different time periods of their early development. The data are presented as mean ± SEM.

**Supplementary Table 2**


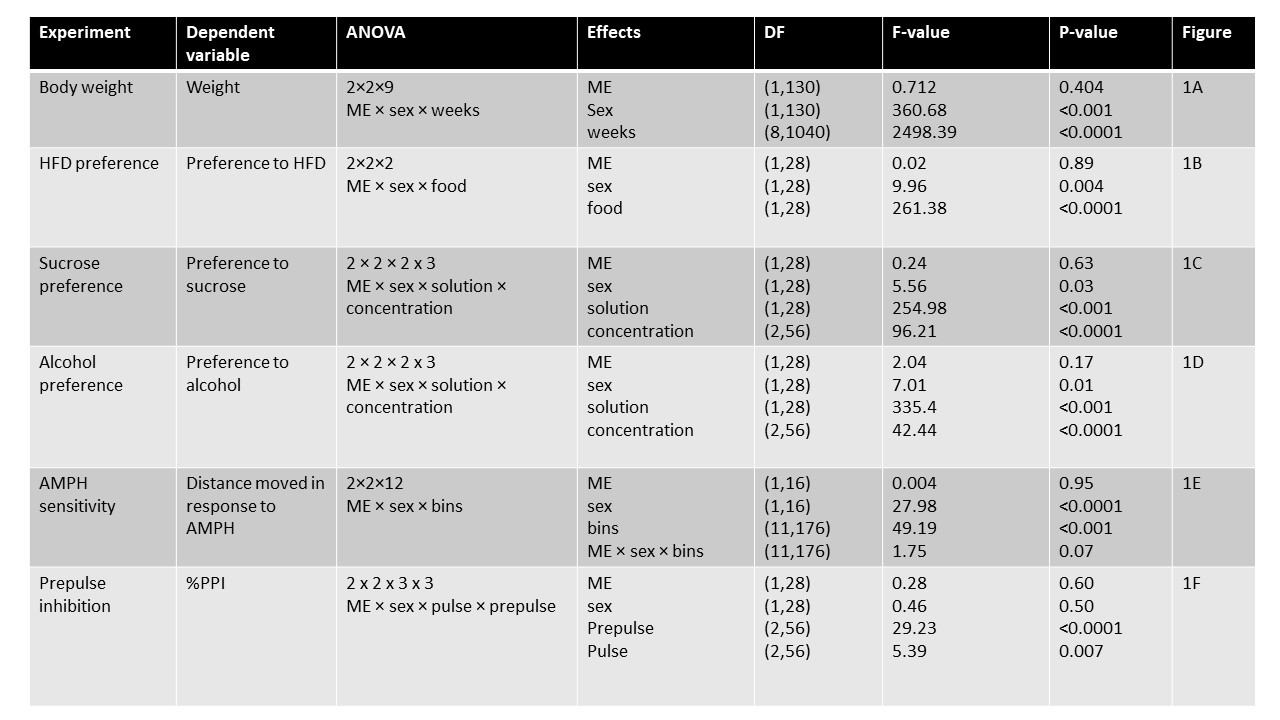


**Table S2:** Summary of the statistics of the experiments conducted in the preconception group. The table shows the dependent measures for each test and summarizes the main effects and interactions between subject’s factors and additional independent factors for each specific experiment. Statistical significance set when P< 0.05. ME = maternal exposure, DF = degree of freedom, AMPH = amphetamine, PPI = prepulse inhibition.

**Supplementary Table 3**

**
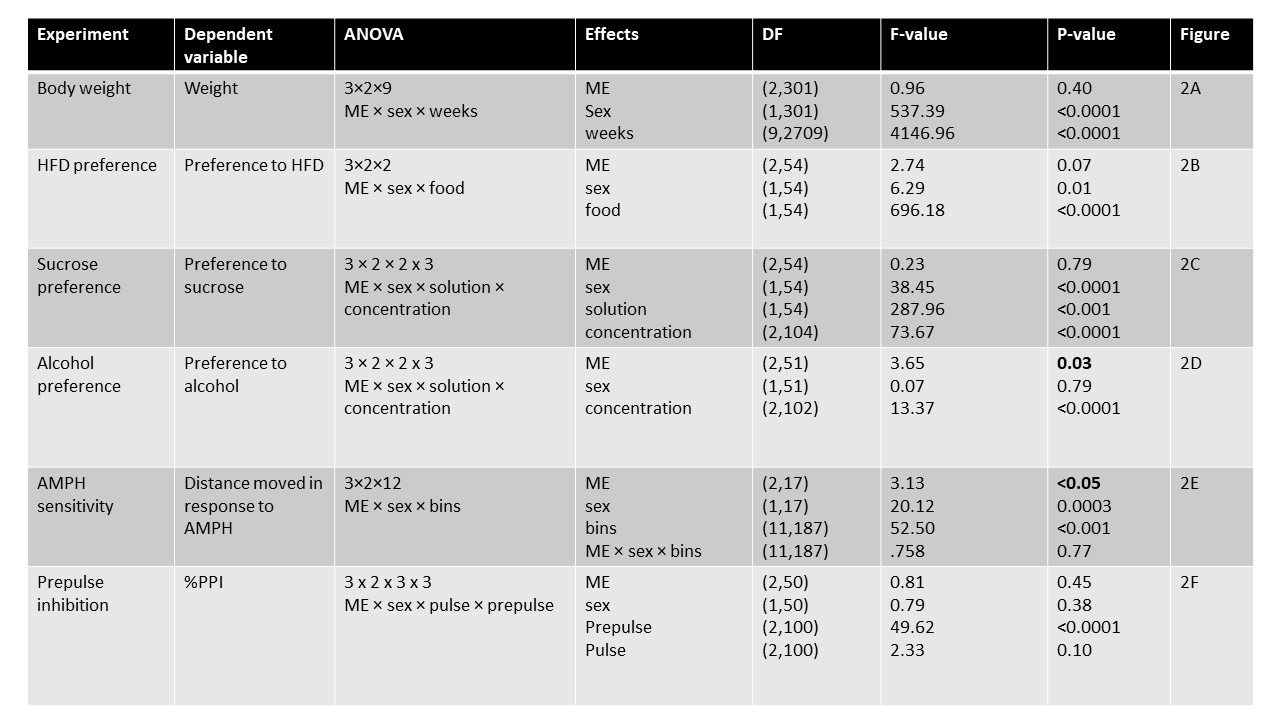
**

**Table S3:** Summary of the statistics of the experiments conducted in the semester group. The table shows the dependent measures for each test and summarizes the main effects and interactions between subjects’ factors and additional independent factors for each specific experiment. Statistical significance set when P< 0.05. ME = maternal exposure, DF = degree of freedom, AMPH = amphetamine, PPI = prepulse inhibition.

**Supplementary Table 4**

**
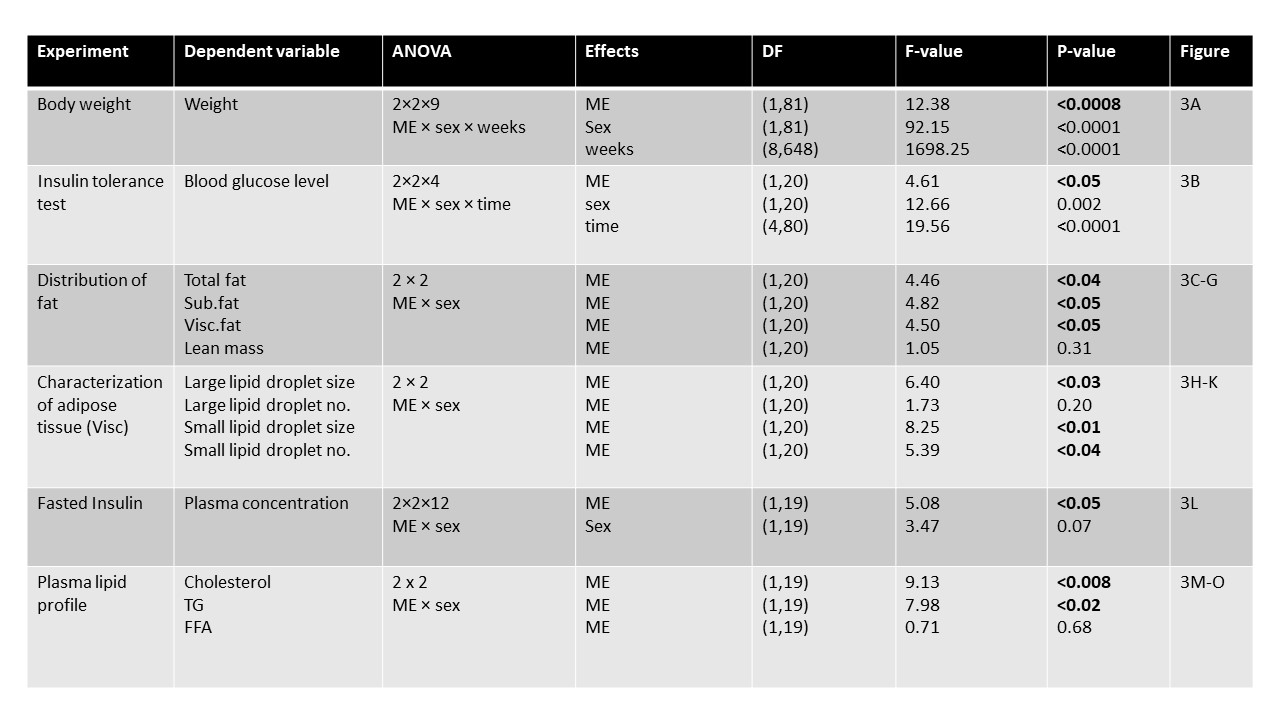
**

**Table S4:** Summary of the statistics of the metabolic experiments conducted in the lactation group. The table shows the dependent measures for each test and summarizes the main effects and interactions between subjects’ factors and additional independent factors for each specific experiment. Statistical significance set when P< 0.05. ME = maternal exposure, DF = degree of freedom, Sub = subcutaneous, Visc = visceral, TG = Triglycerides, FFA = free fatty acids.

**Supplementary Table 5**

**
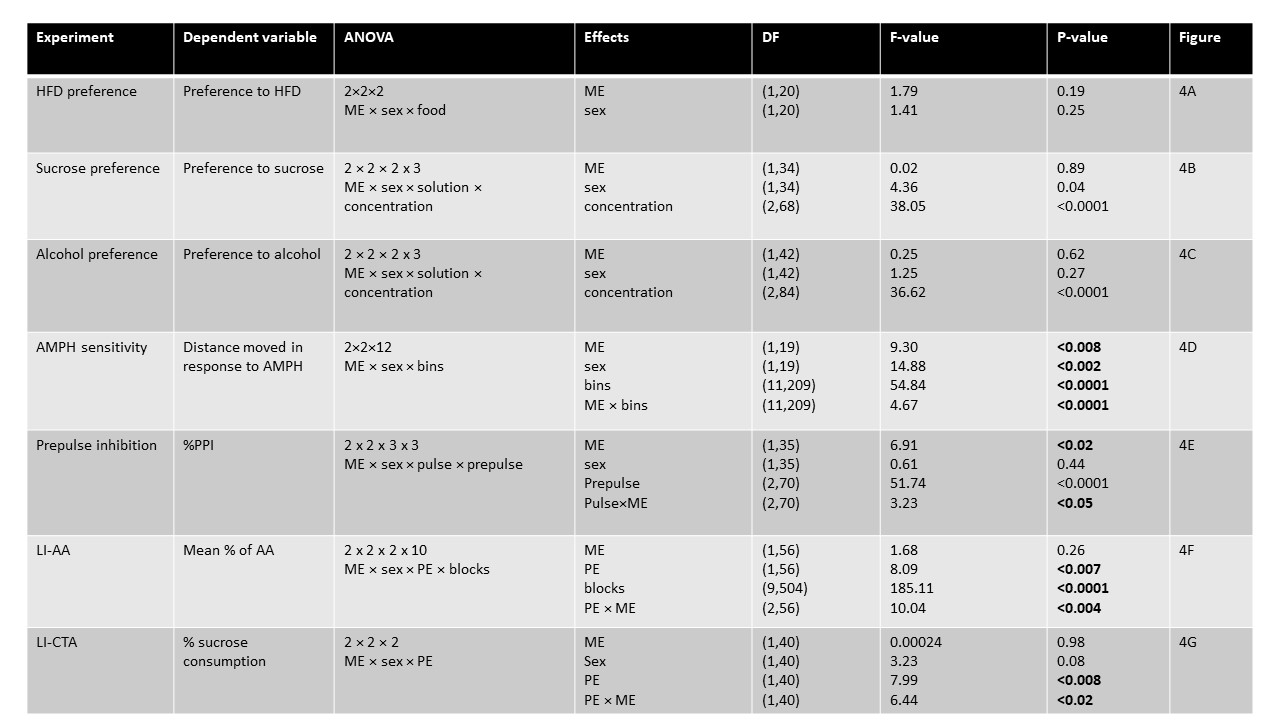
**

**Table S5:** Summary of the statistics of the behavioral experiments conducted in the lactation group. The table shows the dependent measures for each test and summarizes the main effects and interactions between subjects factors and additional independent factors for each specific experiment. Statistical significance set when P< 0.05. ME = maternal exposure, DF = degree of freedom, AMPH = amphetamine, PPI = prepulse inhibition, AA = active avoidance, CTA = conditioned taste aversion.

**Supplementary Table 6**


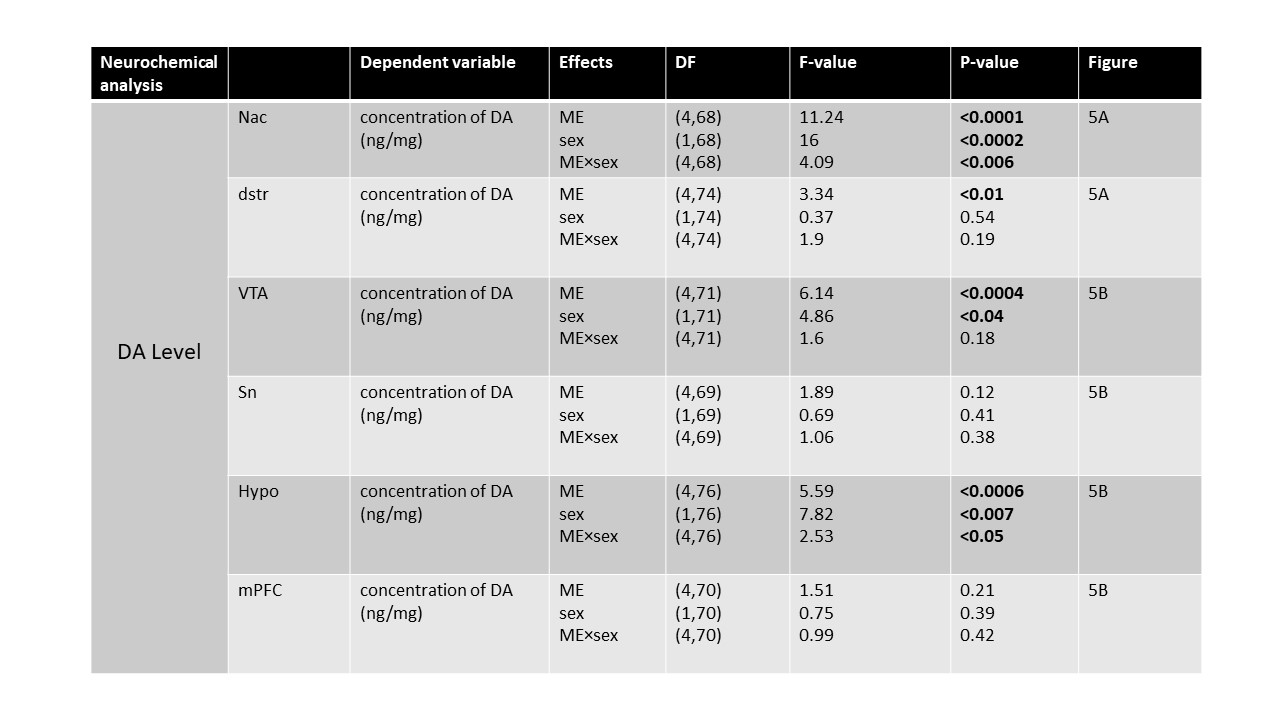


**Table S6:** Summary of the statistics of the dopamine level measure in the different brain regions in the offspring groups. The table shows the dependent measures for each test and summarizes the main effects and interactions between subjects’ factors and additional independent factors for each specific experiment. Statistical significance set when P< 0.05. ME = maternal exposure, DF = degree of freedom, DA = dopamine, Nac = nucleus accumbens, dstr = dorsal striatum, VTA = ventral tegmental area, Sn = substantia nigra, Hypo = hypothalamus, mPFC = medial prefrontal cortex.

**Supplementary Table 7**

**
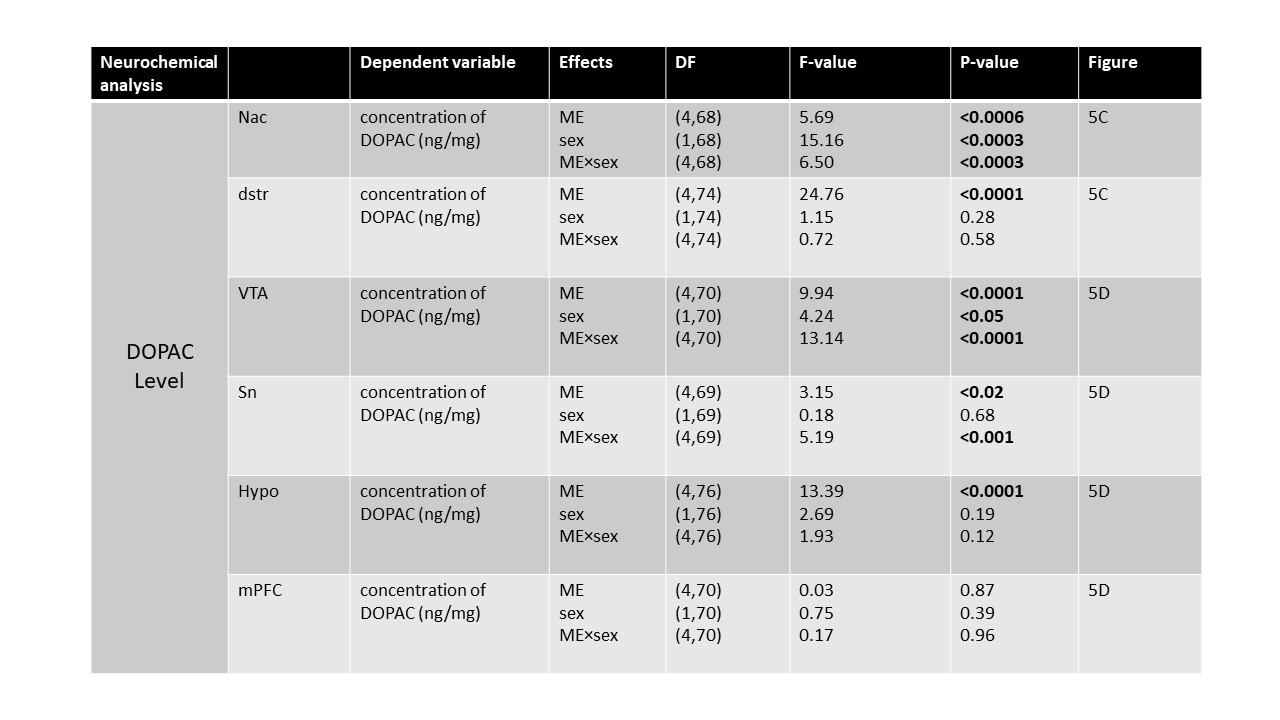
**

**Table S7:** Summary of the statistics of the DOPAC level measure in the different brain regions in the offspring group. The table shows the dependent measures for each test and summarizes the main effects and interactions between subjects’ factors and additional independent factors for each specific experiment. Statistical significance set when P< 0.05. ME = maternal exposure, DF = degree of freedom, DOPAC = dihydroxyphenylacetic acid, Nac = nucleus accumbens, dstr = dorsal striatum, VTA = ventral tegmental area, Sn = substantia nigra, Hypo = hypothalamus, mPFC = medial prefrontal cortex.

**Supplementary Table 8**

**
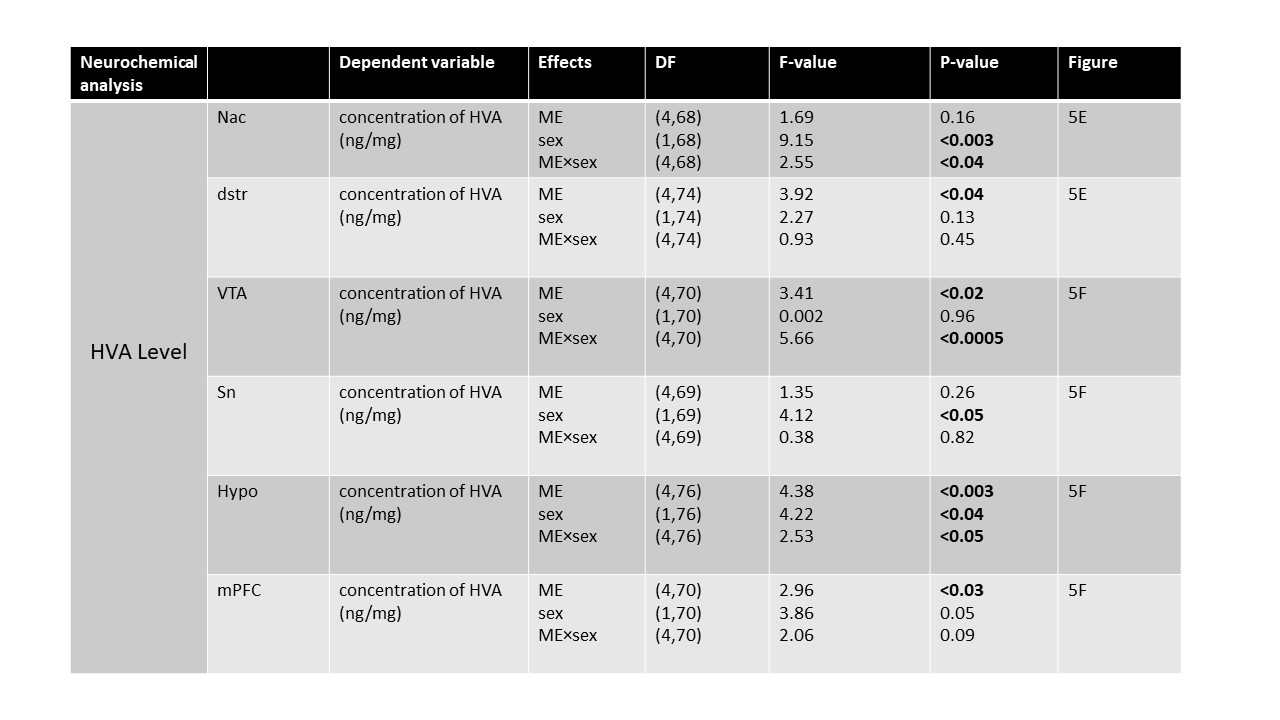
**

**Table S8:** Summary of the statistics of the HVA level measure in the different brain regions in the offspring group. The table shows the dependent measures for each test and summarizes the main effects and interactions between subjects’ factors and additional independent factors for each specific experiment. Statistical significance set when P< 0.05. ME = maternal exposure, DF = degree of freedom, HVA = homovanillic acid, Nac = nucleus accumbens, dstr = dorsal striatum, VTA = ventral tegmental area, Sn = substantia nigra, Hypo = hypothalamus, mPFC = medial prefrontal cortex

**Supplementary Table 9**

**
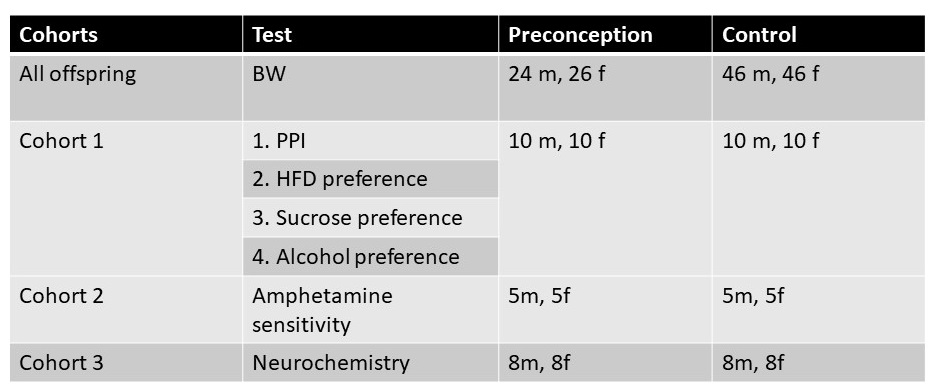
**

**Table S9:** Summary of the number of the offspring in each experiment and the sequence of the tests conducted in the preconception and control offspring.

| **Cohorts** | **Test** | **Early Gestation** | **Late Gestation** | **Control** |
| --- | --- | --- | --- | --- |
| All offspring | BW | 91 m, 76 f | 43 m, 37 f | 32 m, 36 f |
| Cohort 1 | 1. PPI | 10 m, 10 f | 10 m, 10 f | 10 m, 10 f |
|  | 2. HFD preference | 10 m, 10 f | 10 m, 10 f | 10 m, 10 f |
|  | 3. Sucrose preference |  |  |  |
|  | 4. Alcohol preference |  |  |  |
| Cohort 2 | Amphetamine sensitivity | 4 m, 4 f | 4 m, 4 f | 4 m, 4 f |
| Cohort 3 | Neurochemistry | 8 m, 8 f | 8 m, 8 f | 8 m, 8 f |

**Supplementary Table 10**

**Table S10:** Summary of the number of the offspring in each experiment and the sequence of the tests conducted in the gestation and control offspring.

**Supplementary Table 11**

**
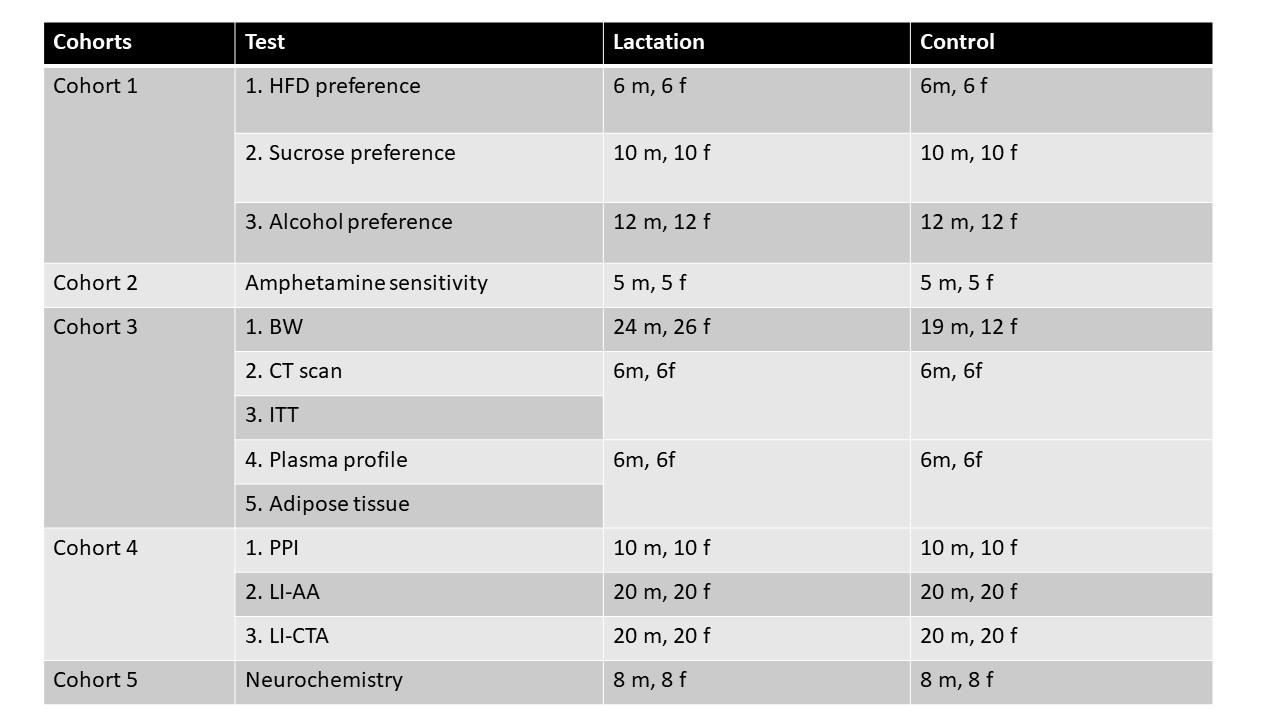
**

**Table S11:** Summary of the number of the offspring in each experiment and the sequence of the tests conducted in the lactation and control offspring.
